# Combining Plant Proteins to Achieve Amino Acid Profiles Adapted to Various Nutritional Objectives—An Exploratory Analysis Using Linear Programming

**DOI:** 10.3389/fnut.2021.809685

**Published:** 2022-02-03

**Authors:** Laurianne Dimina, Didier Rémond, Jean-François Huneau, François Mariotti

**Affiliations:** ^1^Université Paris-Saclay, AgroParisTech, INRAE, UMR PNCA, Paris, France; ^2^Université Clermont Auvergne, INRAE, UMR UNH, Clermont-Ferrand, France

**Keywords:** indispensable amino acids, linear optimization, plant-based protein isolate, protein blend, amino acid profile, cardiometabolic health

## Abstract

Although plant proteins are often considered to have less nutritional quality because of their suboptimal amino acid (AA) content, the wide variety of their sources, both conventional and emerging, suggests potential opportunities from complementarity between food sources. This study therefore aimed to explore whether, and to what extent, combinations of protein ingredients could reproduce an AA profile set as a nutritional objective, and to identify theoretical solutions and limitations. We collected compositional data on protein ingredients and raw plant foods (*n* = 151), and then ran several series of linear optimization to identify protein ingredient mixes that maximized the content in indispensable AA and reproduced various objective profiles: a “balanced profile,” based on AA requirements for adults; “animal profiles” corresponding to conventional animal protein compositions, and a “cardioprotective profile,” which has been associated with a lower cardiovascular risk. We assumed a very good digestibility of plant protein isolates. As expected, obtaining a balanced profile was obvious, but we also identified numerous plant protein mixtures that met demanding AA profiles. Only for particularly demanding profiles, such as mimicking a particular animal protein, did solutions require the use of protein fractions from more specific sources such as pea or canola. Optimal plant blends could mimic animal proteins such as egg white, cow milk, chicken, whey or casein with a similarity reaching 94.2, 98.8, 86.4, 92.4, and 98.0%, respectively. The limiting constraints were mainly isoleucine, lysine, and histidine target contents. These different solutions offer potential for the formulation of mixtures adapted to specific populations or the design of plant-based substitutes. Some ingredients are not commercially available but they could be developed.

## Introduction

Western countries are now entering a new nutritional transition phase that aims to rebalance the dietary plant:animal protein ratio. The development of animal protein substitutes has accelerated in recent years with the formulation of meat analogs and dairy substitutes, making plant proteins promising ingredients (1); their use to achieve adequate protein nutrition is also a key issue in developing countries.

However, plant proteins are often considered as being of poor nutritional quality, mainly because of their lower content in indispensable amino acids, some of which being particularly low in one amino acid, namely lysine, when compared to animal protein or amino acid requirement profiles. This is reflected by poorer values when the quality of these proteins is assessed using methods based on the concept of limiting amino acids, such as the Protein Digestibility Corrected Amino Acid Score (PDCAAS) or the more recently recommended Digestible Indispensable Amino Acid Scores (DIAAS) (2).

However, current methods used to assess protein quality do not easily take account of the possibilities of complementarity between multiple protein sources (3). Indeed, combining two protein sources with low PDCAAS can provide balanced and sufficient quantities of all AA that will cover AA requirements. Furthermore, beyond this basic nutritional objective, it may be advocated that animal proteins have higher nutritional value in specific populations such as the elderly, because of their greater anabolic capacity, particularly during the postprandial period (4). For instance, despite having the same PDCAAS value, soy protein isolate and whey protein differentially stimulate muscle protein synthesis in older humans (5) at rest and following exercise (6). Likewise, in older men, wheat protein requires a greater amount of protein in the meal to overcome a weaker myofibrillar protein synthetic response (7). Therefore, combining plant proteins in order to mimic the amino acid profile of animal protein may be relevant for specific nutritional strategies (8). Lastly, unlike animal protein, plants also contain higher levels of some AA that have been identified as being potentially beneficial to health. This is particularly the case for arginine, cysteine, glutamine, and glycine (9, 10). Here again, mixing protein sources could offer a means to define specific amino acid profiles.

A recent study (11) assessed the amino acid composition of several protein ingredients and characterized a large panel of plant and animal-based protein isolates in order to identify which among the latter were commercially available and are of high anabolic potential. Indeed, data in the literature support the possibility of defining new plant-based protein blends that would be comparable to most animal-based protein sources in terms of their indispensable AA contents, or could be tailored to specific amino acid profiles for use in targeted populations (8). One study identified food pairings from a plant-based food database and the quantities of these foods necessary to meet indispensable AA requirements (12).

Here, our aim was to broaden this approach and identify optimized blends of plant proteins closely reflecting the typical characteristics of different specific amino acid profiles [including the WHO amino acid requirements profile (13, 14)], animal-based protein profiles, and a “cardioprotective profile” that has been associated with a lower cardiovascular risk (15). We then characterized these solutions and the drivers of the linear program in order to deepen our understanding regarding the potential of plant protein sources for human nutrition.

## Materials and Methods

### Database of Plant Protein Ingredients

In order to build a large database of plant ingredients, we screened commercially available plant-based isolates and foods for which complete indispensable AA compositions were available and then postulated that protein isolation was technically possible. We used amino acid compositions from the USDA database (16) and from the data sheets supplied by protein ingredient producers. From the USDA food database, we selected foods labeled as Raw, Plant-based or Dried when available. The following food groups are represented: “legumes and legume products;” “Fruit and fruit juices;” “Vegetables and Vegetable Products;” “Spices and Herbs;” “Nut and Seed Products;” “Cereal Grains and pasta.” Finally, we retained in the database the compositions of 151 plant protein ingredients only because of the limited data available on the amino acid composition of foods. Amino acid contents are expressed as g per g of protein in 100 g of product on the USDA base; this was also the case on the technical sheets. Because crude protein contents (*N* × 6.25) and “true” proteins (i.e., the sum of amino acids although it does not consider the weight of prosthetic groups) vary according to the total protein content (17), we chose to standardize the amino acid contents per 100 g of total amino acids.

### Linear Programming

A linear optimization program (Linear Programming) is designed to maximize or minimize a linear application, called an objective function, on a set of constraints in the form of linear equations or inequalities. This mathematical tool is an ancient method developed that has been applied to the nutritional field for the first time in (18) to find the optimal diet at minimum cost. Since then it has been broadly used to explore solutions to nutritional problems in different contexts (19).

We used the LP simplex method (Microsoft Excel Solver tool). The program assigns values to variables iteratively according to the Simplex algorithm in order to identify solutions; i.e., that satisfy all the constraints. When there is no solution, the method consists in degrading the constraints as little as possible (which is referred to as “goal programming”).

Linear programming does not result in a list of solutions ranked in descending order from the optimal solution; only the optimal solution is identified. In order to further explore the domain of the solutions, we removed from the database, iteratively and one by one, the ingredients composing the optimal mixture, according to different orders, and then ran the linear program again, leading to another solution that was newly optimal but suboptimal when compared to the previous one. We reiterated this process until no solution was found, i.e., no combination could satisfy all the constraints.

### Constraints and Objective Function Used for Linear Programming

We chose to standardize the protein amount at 30 g, so as to maintain a dose that is referred to as a protein-rich meal and is in line with a potential dietary application. In detail, the rationale for the constraints was based on three expected features of the protein blend. First, the portion of protein supplied by the blend had to remain similar to current dietary habits; 30 g represents the average protein intake per meal (20, 21) and the amount of protein powder in a typical serving size scoop. Second, in elderly people, 30 g represents the amount of good quality protein required to overcome the “anabolic threshold” (22). Finally, although recent findings have tended to demonstrate that muscle protein synthesis is not driven by the Indispensable Amino Acid (IAA) quantity in a meal as such, but rather by the meal's leucine content (23, 24), we chose to set the protein at 30 g in the linear program in order to address complementary goals.

The AA contents of the protein sources were therefore expressed in g/30 g of AA in the database. The different objective functions and constraints were then applied according to the chosen objectives and related AA profiles. As a result, the linear program operated on the proportion of each database protein ingredient to be incorporated in the protein blend. Thus, the sum of the quantities was equal to 100% of the real protein mass of the final protein ingredient, i.e., 30 g. The objective function used in this problem (25) was maximization of the sum of the contents in indispensable AA. The variables were the proportions of each of the protein sources to be included in the blend (from 0 to 100%). The constraints related to the quality of the blend, meaning that the values in each indispensable AA needed to be greater than or equal to the values defined by the profiles taken as the objective.

We applied various constraints in the linear program in order to test the degree to which plant protein blends could mimic various amino acid objective profiles. These profiles were the “WHO profiles” for adults and 0–6-month-old infants, numerous “animal” profiles and a “cardioprotective” profile.

Under the “WHO profile” the optimized protein blend met total body IAA requirements (13, 14) (Supplementary Table 1).

For the “animal” profiles, we studied numerous animal products, including beef, veal, lamb, pork, rabbit, chicken, turkey and duck meats; egg white; cow, sheep and goat milks and widely consumed (26) protein fractions such as casein and whey (Supplementary Table 2).

For the “cardioprotective” profile, we used the structures (factors) of the amino acid intake profiles that were associated with cardiovascular mortality in a previous study (15).

In Tharrey et al. (27), 18 protein food groups were defined according to their origin and their contribution (in percentage) to total protein intake. Then five protein dietary patterns (factors) were identified using factor analysis (PCA and varimax rotation methods). Factors were principal components, defined as a linear combination of food groups weighted by loadings. In cox proportional hazard regressions models, meat based and nuts based dietary patterns were positively and negatively associated with cardiovascular health (and CVD mortality) when adjusting for several confounders [age, sex, race, energy intake, BMI, physical activity, smoking status, alcohol consumption, education, income, marital status, non-vegetarian; semi-vegetarian; pesco-vegetarian; lacto-ovo-vegetarian; vegan status, saturated fatty acids, unsaturated fatty acids, fiber, sodium, vitamin B6, B12, folates, antioxidants (vitamins A, C, E), fat from meat products (fish excluded), and fat from nuts]. This association between protein dietary patterns and CVD could be mediated by their respective AA contents and/or other substances (e.g., polyphenols) tightly associated with protein in the plant matrix.

In Tharrey et al. (15) the same approach was adopted than in the previous study except the contribution (in percentage) of the 18 proteinogenic AA to the total protein intake was used. Then three AAs dietary patterns were identified using factor analysis (PCA and varimax rotation methods) and AA factors were principal components, defined as a linear combination of AA weighted by loadings. In cox proportional hazard regressions models, factor 1 and factor 3 were associated with cardiovascular health when adjusting for several confounders (listed above) plus the five protein dietary patterns identified in (27) to decipher the specific effect of AAs dietary patterns effects from that of the protein package.

- AAs dietary pattern 1 (or factor 1), with high loadings of indispensable AA such as branched chain amino acids, lysine and methionine, was independently and positively associated with cardiovascular disease (CVD) mortality.
- AAs dietary pattern 3 (or factor 3), with high loadings of non-indispensable AA, was independently and negatively associated with CVD mortality.

Only AA strongly associated with factors 1 and 3 were considered (standardized component loads >0.7) in the present study. These factors were defined as follows:


$$\begin{array}{l} factor1=0.78\;thr+\;0.91\;ile\;+\;0.84\;leu\;+\;0.95\;lys\\ \text{​}\;0.93\;met\;+\;0.83\;tyr\;+\;0.86\;val+0.79\;his \end{array}$$

$$\begin{array}{l} factor3=0.92\;arg\;+\;0.83\;asp\;+\;0.85\;gly. \end{array}$$

The objective was to design a plant protein blend that would maximize Factor 3 minus Factor 1.

### Similarity Index

For each optimal solution identified, we chose to evaluate the degree to which the solution met the objective, using a similarity index (SIM) weighted according to the contents in nine indispensable AA, as follows:


$$\begin{array}{l} SIM=\left(\frac{1}{9}\right)\overset{9}{\underset{i=1}{\sum }}Var_{i\;}with\;Var_{i}=\frac{i\;blend\;content}{i\;pattern\;content} \end{array}$$

Where i refers to one of the nine indispensable amino acids. Var_i_ was limited to a maximum of 1.

SIM = 1 means the similarity is 100%, i.e., that the solution is optimal, with all values for each indispensable AA being greater than or equal to the values defined by the profiles set as the objective. Alternatively, if SIM < 1, then the solution does not meet the objective for at least one indispensable AA but the optimized profile and the objective profile have SIM expressed as % as a common part.

### Testing the Robustness of the Linear Program in Terms of the Variability of Compositional Data

Napin contents in rapeseed seeds may vary among varieties depending on the fertilization conditions, with the napin/cruciferine ratio largely dependent on the sulfur/nitrogen ratio (28). The napin isolate (supertein^®^) that we used in the database is obtained from canola (Canadian rapeseed), which is similar to French rapeseed in terms of its erucic acid and glucosinolate contents. By contrast, the AA composition of the napin fraction differs, especially regarding lysine and cysteine.

Therefore, in order to assess the impact of variations in the composition of ingredients on the quality of the solutions generated by linear programming, we selected an AA target profile (beef short ribs) to be replicated using plant proteins only. This particular target profile was chosen because the optimal plant mixture contained a substantial amount of napin (>25%). We then estimated the effect on the amino acid profile of replacing this protein ingredient with another (rapeseed albumin) in the optimal mixture.

## Results

### Variability of Indispensable Amino Acid Contents in Plant vs. Animal Ingredients

When ranking the protein ingredients in the database in terms of their IAA contents, we found that some levels were high in plant-based protein ingredients in particular. This was notably true for tryptophan and phenylalanine, where the 15 richest protein ingredients were of plant origin (Supplementary Table 3). By contrast, lysine, methionine, histidine and isoleucine were only found at low levels in plant-based protein ingredients. In fact, only a few ingredients can compete with animal products regarding these IAA: pea albumin for lysine, Brazil nut for methionine, napin for histidine and Oriental radish for isoleucine. Lastly, some IAA, such as valine, threonine, and leucine, were found at high levels in both animal and plant-based protein ingredients when principal component analysis was performed on our database (see Supplementary Figures 1, 2), suggesting it may be possible to formulate plant blends of potential interest for muscle anabolism.

### Obtaining AA Profiles From Mixed Plant Protein Ingredients That Are Balanced According to the WHO Reference Profile

**Figure 1:**
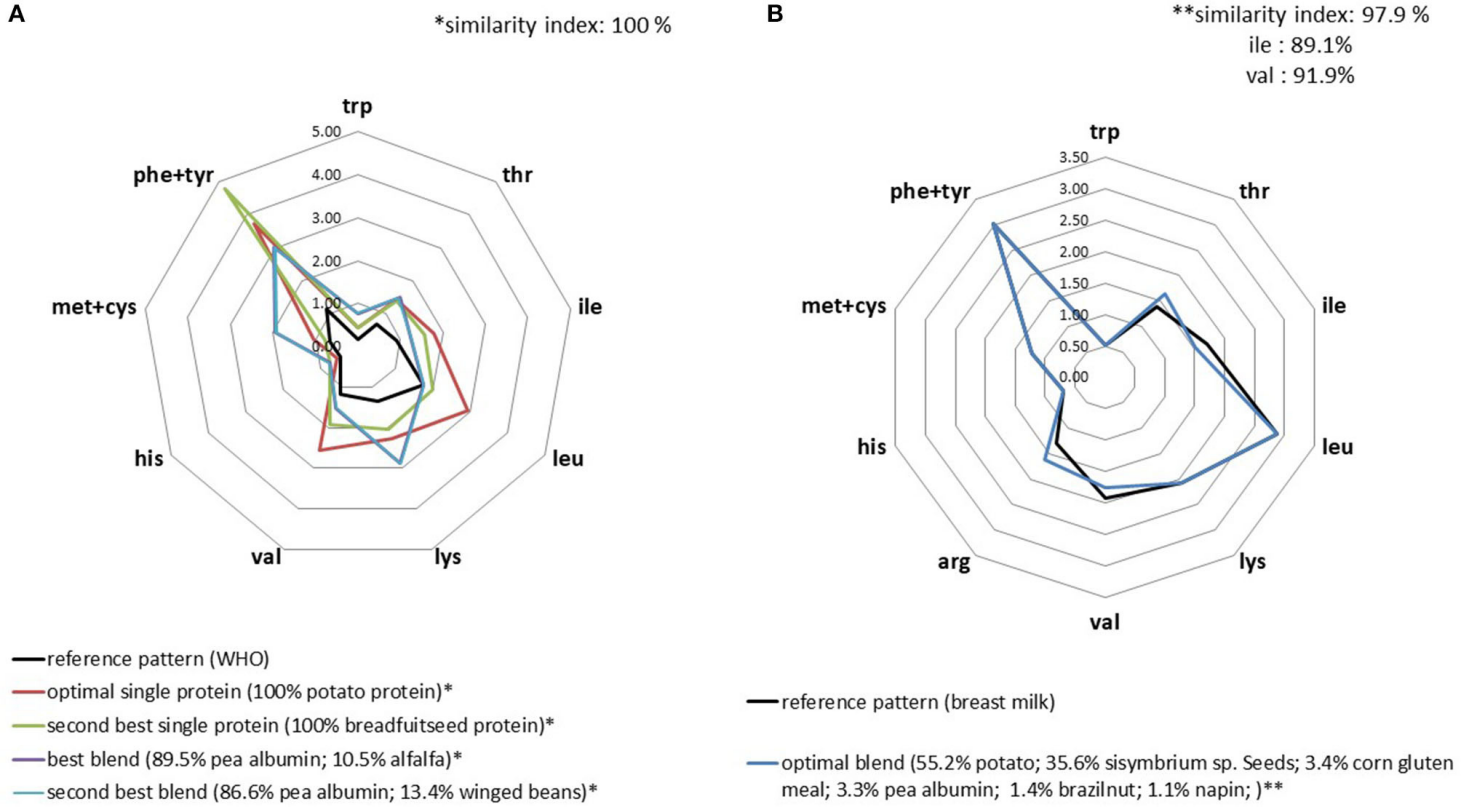
IAA profiles (g/30 g) of the optimal plant protein blends that best replicated the IAA profiles for adult **(A)** and infant **(B)** WHO requirements.

In the database, only potato and breadfruit seed proteins were balanced according to the WHO reference profile (Supplementary Table 1). This means that these proteins could supply sufficient amino acids if they were consumed as the only protein source in the diet and at the low levels necessary to cover standard protein (nitrogen) requirements. Of course, this implies that protein digestibility is satisfying. When considering mixing plant proteins to match the WHO reference profile, we found a very large set of solutions. The optimal blend (i.e., the richest in IAA) was composed of pea albumin and alfalfa (at 90 and 10%, respectively). Alfalfa supplied the extra leucine required to match the level in the target profile, which is not attained by pea albumin alone. As for the IAA requirements of infants, it was more difficult to match plant protein blends with the IAA pattern in breastmilk. We found a similarity index of 97.9% for the best blend composed of six ingredients, but was slightly suboptimal regarding the isoleucine content (Figure 1).

### Mimicking Total Animal Protein and Animal Protein Fractions With Plant Protein Blends

**Figure 2:**
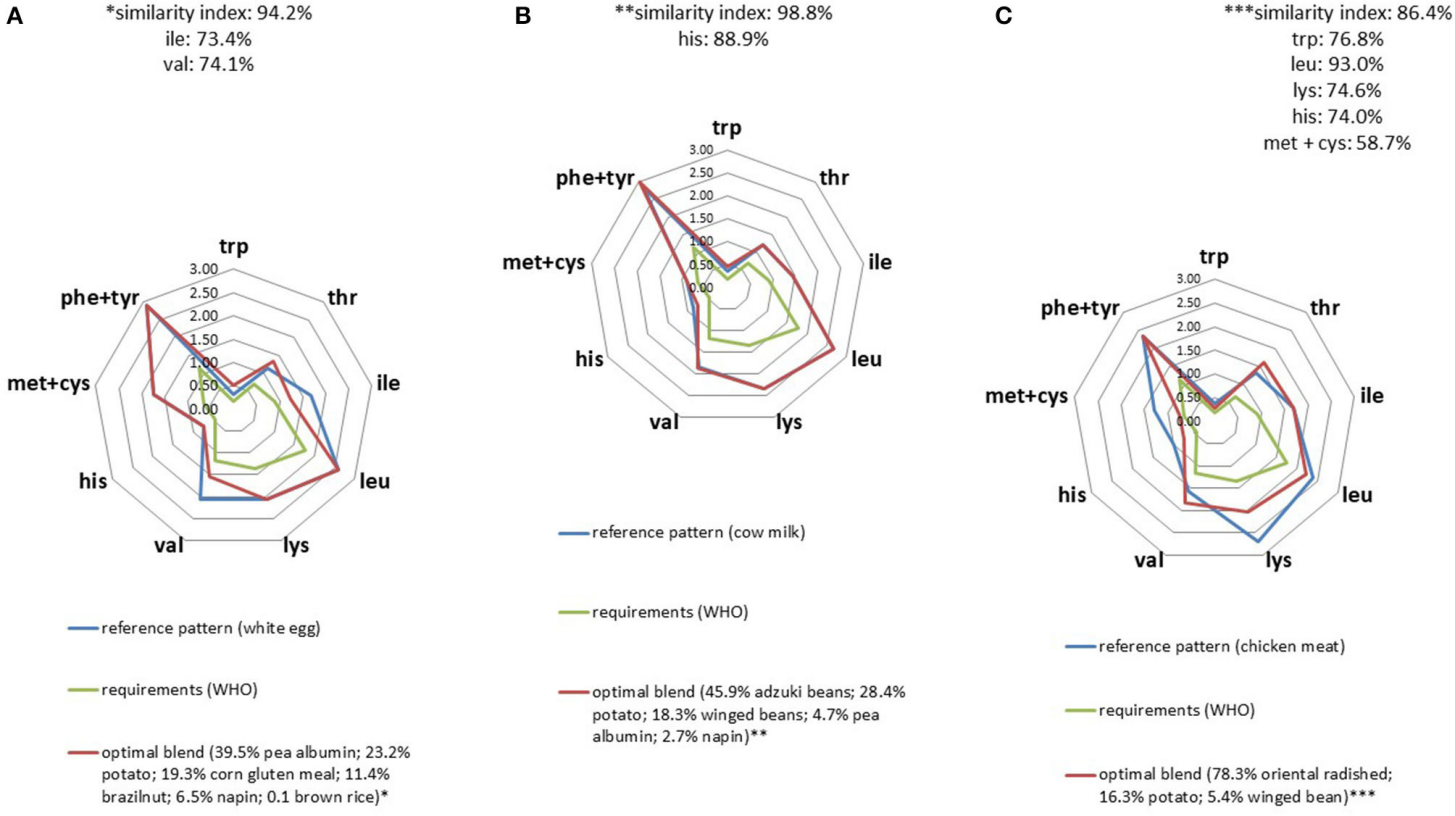
IAA profiles (g/30 g) of optimal plant protein blends that best replicated the IAA profiles of egg white **(A)**, cow milk **(B)**, and chicken meat **(C)**.

For many animal protein profiles, we were able to identify plant blends as optimal solutions that proved to be over 85% similar (Figure 2 and Supplementary Figures 3–11). Optimal plant blends could mimic animal proteins such as egg white, cow milk, chicken, whey or casein at 94.2, 98.8, 86.4, 92.4, and 98.0% respectively, the limiting constraints being mainly the target contents in isoleucine, leucine, and lysine.

**Figure 3:**
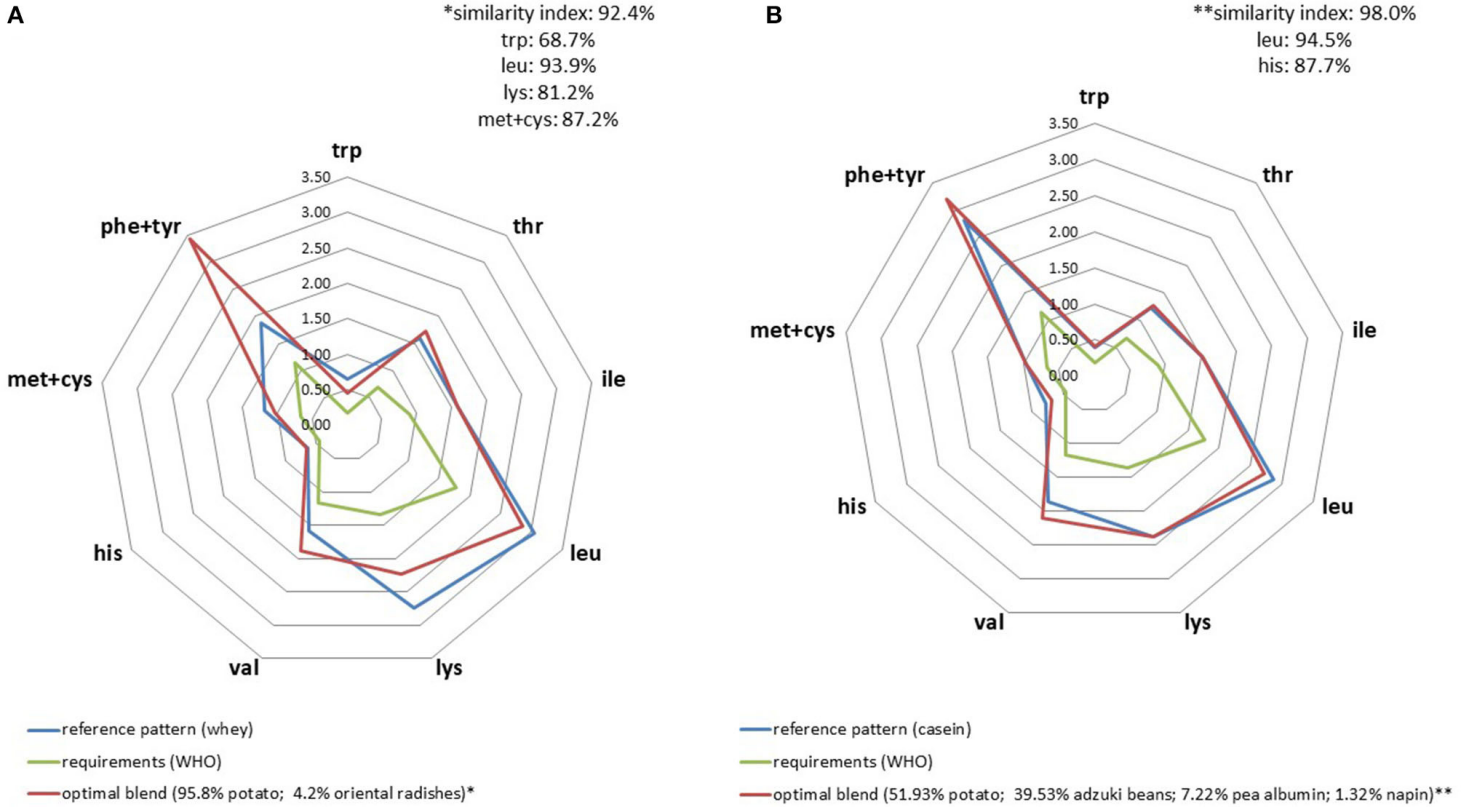
IAA profiles (g/30 g) of optimal plant blends that best replicated whey **(A)** and cow milk casein **(B)** IAA profiles.

When applied to milk protein fractions, AA profiles seemed to be slightly more difficult to mimic using a plant-based protein blend compared to casein (Figure 3) particularly because of its high leucine content. Another element worth noting was the need to incorporate pea albumin fraction in the mix in order to satisfy IAA constraints. The higher the IAA constraints, the higher the proportion of plant protein fraction it was necessary to incorporate in the blend. For instance, the amount of pea albumin required for the whey profile was 59% compared to 11% for casein.

Suboptimal solutions were found for horse meat, with constraints that needed to be lifted for some amino acids, namely histidine, methionine, isoleucine and lysine, and the quality of the optimal plant-based IAA blends varied depending on the meat cuts that were set as target profiles (Supplementary Figure 6).

### Obtaining Plant Blends That Mimic a “Cardioprotective” Amino Acid Profile

**Figure 4:**
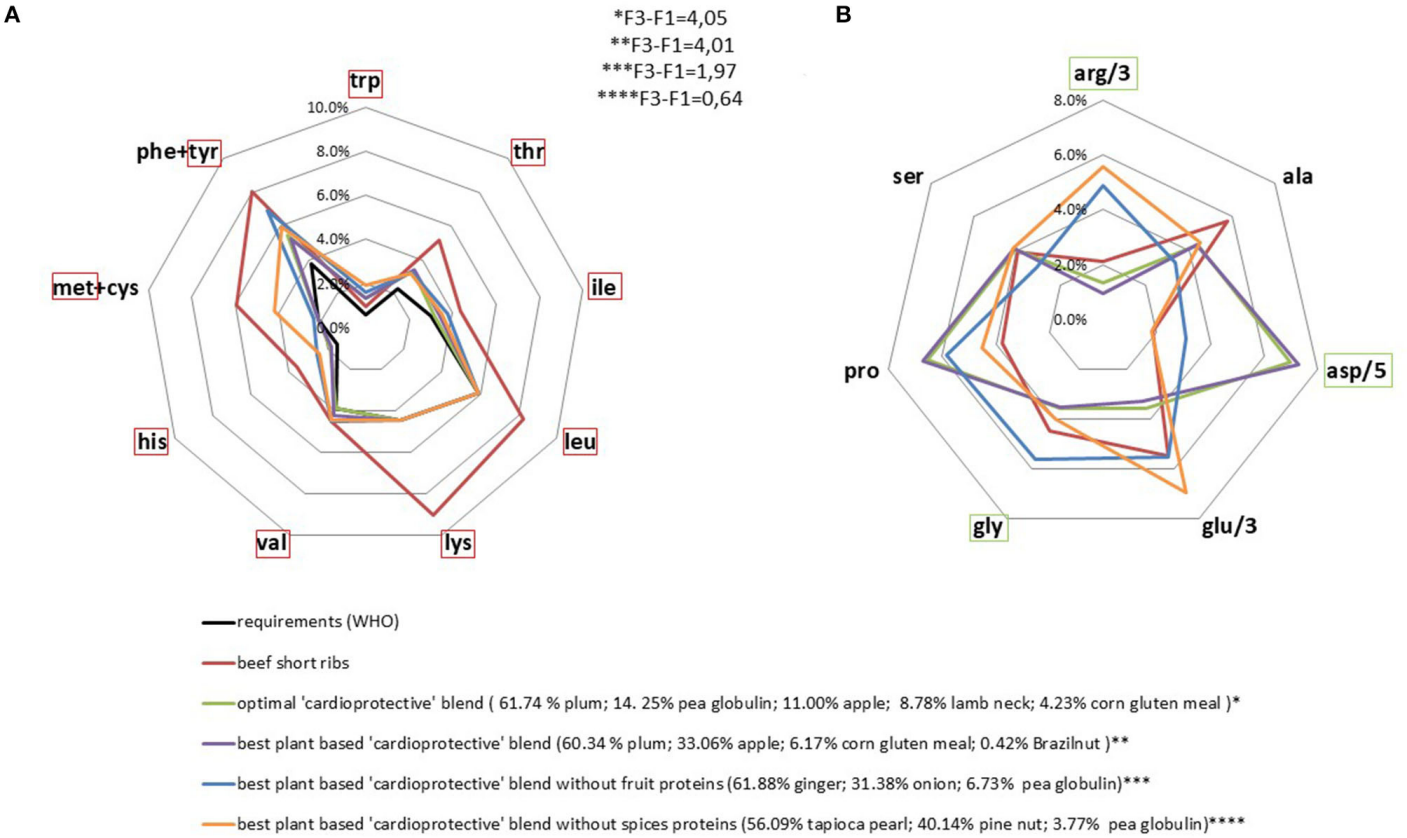
AA profiles (% of protein intake) of optimal protein blends based on a lower cardiovascular mortality risk. Requirements and beef AA profiles are presented for comparison. IAA requirements were set as the minimum content. The indispensable amino acids in the red frame were minimized, yet constrained to be higher than requirement value **(A)** and the non-indispensable amino acids in the green frame were maximized **(B)**.

We found a large panel of solutions that matched the “cardioprotective” profile. Considering both plant and animal protein sources, the optimal blend was >90% plant-based. Removal of the only animal product from the blend had a very limited impact on the result obtained *via* linear programming. This new optimal mixture was 100% plant-based and composed of protein from fruits (apples 33% and plums 61%); corn (6%), and Brazil nuts (<1%) (Figure 4). It was particularly rich in aspartic acid and proline. Inversely, leucine, lysine and methionine were included at the level of nutritional requirements only (Figure 4), in line with their negative weighting in the targeted profile (27). The constraint on the methionine content was active (Table 1) which means that it limited the possible range of solutions to be found during linear programming. This rendered it difficult to maximize the final linear amino acid combination (F3-F1) without resorting to specific plant-based protein sources (fruits and spices), which have the disadvantage of being low in protein or consumed in relatively small quantities as part of a standard diet.

**Table 1:** Active constraints during linear programming, which led to infeasible solutions and to unmet non-active constraints.

**Target AA profile**	Active constraints^*^	**Unmet constraints**
IAA adult requirements (WHO)		/
IAA 0-6 months infant requirements (WHO)	met+cys, ile	ile
“Animal”: cow milk	his, leu, lys	his
“Animal”: whey	leu, lys	leu, lys, trp, met+cys
“Animal”: casein	leu, lys	leu, his
“Animal”: chicken	lys, ile	lys, trp, leu, his, met+cys
“Animal”: white egg	ile, met+cys	Ile, val

*Active constraints needed to be relaxed and the non-compliance to relaxed constraints was minimized by goal programming*.

* *By definition, the weight of the blend is always an active constraint*.

### Active Constraints During Linear Programming

Active constraints during linear programming affected the optimization of the objective function, i.e., prevented to find better solutions. By increasing the quantity of protein in the blend or reducing the IAA target contents (especially lysine and sulfur AA in animal profiles), the optimal solutions could be improved (see Table 1 and Supplementary Figures 3–11 for other target profiles).

### Linear Programming Is Robust in Response to the Variability of Compositional Data

Following a test of robustness on the linear program, we were able to show that using a protein ingredient from a different supplier only marginally modified the IAA profile of the optimal solution (see Supplementary Figure 12), suggesting that the method is not highly sensitive to database uncertainties.

## Discussion

### Main Findings

Using an original approach, we were able to study whether, and to what extent, plant protein could be blended optimally to reproduce or closely approach specific AA profiles. We identified several series of optimal plant protein blends, determining which plant protein ingredients were levers and which amino acids were the most binding in the process. One important finding was that solutions could be found even for particularly demanding profiles such as meat, with optimal mixes being >85% similar to animal profiles and theoretically simple to formulate using between two and five ingredients.

The main limitation of this work is the lack of data concerning protein ingredients digestibility. Firstly, most of the protein isolate in the database are not yet developed so protein digestibility cannot be known. Thus, we assumed that isolation from plant-based foods was technically possible and led to similar amino acid composition although this composition may vary between protein ingredients and the parent material. For instance, the digestibility of rapeseed proteins has been shown to vary according the purification and preparation of the protein isolate (29–31). In addition, the variations may be non-negligible when considering specific amino acids. For instance, the content and composition of AA may be influenced by the method used to extract proteins. This is particularly the case for lysine, where ultrafiltration can reduce lysine losses (30, 32) and lower the levels of undesirable chemical compounds (1) when compared to acid precipitation.

Secondly, native plant proteins may contain many antinutritional factors that hinder digestion (33, 34) (e.g., trypsin inhibitors, saponins, etc.) or have a protein structure resistant to hydrolysis. However, with some exceptions (35), real ileal digestibility studies in humans have reported high digestibility estimates for plant protein isolates (89–92%) that are comparable to those of meat or egg proteins (90–94%) (3). Indeed, isolate extraction processes can reduce or suppress the activity of antinutritional factors (36–38) and preparation processes (such as heating) can often markedly improve protein resistance to hydrolysis *via* protein denaturation (29). Protein isolations methods such as ultrafiltration are been developed to increase protein solubility (31) so as to have technologically functional protein ingredients but also to produce protein with high digestibility.

Finally, there are indeed many parameters that can influence protein composition, digestibility and functionality, thus influencing the results of the present study. This is critical for lysine and leucine, which often constrained the solutions. Therefore, it would be a worthy aim to correct for differences in amino acid ileal digestibility in future work of this nature.

### Protein Ingredients Used and the Most Sensitive AA

It is interesting to note that the plant protein ingredients selected were often the same whichever animal protein was taken as the target profile (see Supplementary Material). We were able to identify key plant protein ingredients on which satisfying the target objective of AA composition was based, particularly for animal protein patterns. These theoretical key protein ingredients were: legumes (lima beans, pea albumin and winged beans protein isolates), starchy vegetables and vegetables (potato protein and oriental radishes isolate), cereal grains (corn and brown rice protein isolates), fruits (persimmon protein isolate), and nuts and seeds (napin: canola albumin, sisymbrium seeds and Brazil nut protein isolates). These results were consistent with a previous study that had suggested potato and pea proteins as the most promising plant-based complementary sources to achieve high quality blends (39). Taken together, these ingredients offered a series of amino acid profiles rich in some amino acids that proved to complement each other in order to meet animal amino acid profiles. Indeed, potato, persimmon, lima beans, winged beans, and sisymbrium seed protein isolates are particularly rich in BCAA. Napin protein isolate is extremely rich in cysteine and was used in the plant blends to increase the sulfur amino acid content; it is also rich in histidine. Corn protein isolate is especially rich in leucine. Brown rice protein isolate is also rich in leucine and additionally in valine.

As far as amino acids are concerned, and as we will further discuss, five amino acids appeared to operate often as active constraints in the linear program: histidine, lysine, sulfur amino acids (methionine), isoleucine, and leucine.

### Lessons for Public Nutrition

In addition to producing a mixture that conveyed all IAA in the expected amounts, the results presented here could be interpreted as indicating the complementarity of proteins in the overall diet, as in plant-based diets. To reach the standard requirement for IAA, it is not necessary for the profile to be reproduced in a single meal. It is usually considered that proteins should complement each other over a longer period, classically ~1 day (40). As we have shown here, there are many ways that plant proteins can be mixed to provide all IAA at the concentration required for a standard balanced profile corresponding to requirements. Historically, cereals and beans have long been considered as offering simple complementation, because cereals are low in lysine and beans in sulfur amino acids. However, the level of sulfur amino acids in plants is not a problem, because levels are indeed high in most plant proteins and only marginally low in legumes. The results presented here further confirm that sulfur amino acids are not limiting on practical grounds in terms of meeting standard requirements. Ultimately, the really critical amino acid is lysine, as we found during our study when trying to identify a plant mix based on the reference WHO profile, and had reported in our earlier work modeling the increase in the share of plant protein in the general population (41). It should be borne in mind that lysine is critical when the amount of protein is constrained in the meal (as in the present work where a single protein dose was defined) or the diet (as in the reference WHO profile, which supposes a protein intake equal to the protein requirement). Otherwise, concerns regarding lysine are obviously alleviated by higher amounts of protein, such as those consumed in economically developed countries. Finally, if taken individually, plant protein is generally of lower nutritional quality than animal protein, usually because of its unbalanced amino acid content, but our data show that the complementarity of plant proteins, which is simple to ensure, can offer competitive alternatives to animal protein sources, even at very low level of protein intake.

### Defining Protein Blends for Meals With Specific Applications

Data suggest that protein utilization efficiency is lower in older adults (35 vs. 48% in younger adults) (42) and resistance to muscle protein anabolism increases with age, thus limiting the positive effects of dietary proteins on muscle protein synthesis (43). One way to overcome this “anabolic resistance” consists in increasing the overall consumption of dietary proteins/IAA beyond required levels (44, 45) to 0.9–1.0 g/kg per day, and concentrating protein consumption in fewer food intakes (42). Several studies have tried to define the optimal protein intake per meal that would be sufficient to overcome the anabolic threshold and thus generate a maximal anabolic response in the elderly. It has been reported that the optimal intake to maintain muscle protein synthesis, and presumably long-term mass and function, is ~30–35g per meal (45) of high-quality protein in elderly adults. This represents a protein consumption of 0.40 g/kg/meal (45), or about 15 g IAA/meal (25, 45).

Although mimicking animal proteins is not necessary to cover IAA requirements, such a policy could be beneficial in terms of their anabolic properties in specific populations such as the elderly. But as shown here, it is possible to identify several plant-based blends that could result in mimicking animal proteins.

As well as lysine, other IAA proved to be active constraints; i.e., they constrained the solutions. These critical IAA varied depending on the target animal profile, being histidine in some cases and sulfur amino acids in others. Branched-chain amino acids (BCAA) were constraining in many cases. This is important because BCAA, and particularly leucine, are considered to drive the anabolic potential of animal protein. Furthermore, it appeared more difficult to mimic whey from plant protein because of the very high leucine level in this highly specific animal protein fraction. However, we identified numerous plant protein blends which in a single dose (30 g) contained sufficient leucine (3 g) (46) to elicit postprandial protein synthesis in the elderly. This work thus offers a proof of concept that plant protein can be mixed to mimic animal protein for specific uses, and can reach the amount of leucine that is considered sufficient to overcome postprandial anabolic resistance. For this to be implemented in real formulations, the challenge remains to source plant protein that would be available as a purified ingredient to enable the production of realistic meals containing high levels of protein.

While one might have anticipated problems in mimicking animal protein, we were not in fact surprised that plant protein could perfectly reproduce the “cardioprotective” AA profile chosen as a reference for this study. Indeed, this amino acid profile has been associated with a plant protein profile, and both profiles are associated with lower cardiovascular mortality (27). Because of its two components, this AA profile has been negatively associated with protein from meat and processed foods (which were mostly animal-based) and positively associated with grains, legumes, fruits and vegetables, and nuts and seeds. This reference profile is rich in aspartic acid (and/or asparagine), glycine and arginine, which are classified as dispensable amino acids because they are not required in the diet regarding protein synthesis (47). This reference profile contains moderate amounts of BCAA, lysine and methionine, inasmuch as they were set at the standard levels to achieve requirements (WHO reference profile). Therefore, constraints on obtaining an optimal blend mainly concerned a sufficient content in sulfur amino acids and high levels of the three dispensable AA (including arginine). Sufficient lysine levels did not prove problematic when substantial quantities of plant protein were mixed together (30 g).

### Deficiencies and Uncertainties Regarding AA Contents in Plant Protein Ingredients

The albumin fraction of rapeseed protein (napin) and the globulin fraction of pea protein often appear to be required to achieve a targeted IAA composition, especially with regard to lysine. However, the AA compositions of the protein fraction of most of the other plant sources were lacking so they could not be fully integrated in the ingredient database. It is anticipated that protein fractions from other sources may be of use to find other solutions, which could be similar or possibly better. Furthermore, because the data came mainly from the USA and compositions vary between cultivars (48, 49), we might have found different optimal solutions if we had used data involving different varieties grown in other regions.

### Next Steps, Development, and Perspectives

Because of the versatility of the approach adopted here, it will be possible to expand and improve the database in order to find better and newer solutions, and it may also be possible to adapt the approach to specific contexts (such as agroecological variations) and specific populations [such as severe/moderate acute malnutrition, for instance (50, 51)]. This approach could provide valuable guidance to manufacturers when developing products adapted to the shift toward plant-based diets, and identifying innovative plant protein sources. However, in order to ensure these potential applications at an industrial scale, the approach requires additional information regarding the availability and identification of appropriate extraction techniques for protein ingredients. Indeed, among the theoretical ingredients that we used, very few are available on the market except for potato protein (Solanic^®^ Avebe) and rapeseed albumin (Supertein^®^ Burcon), both of which are promising in terms of their nutritional and functional properties. Thus, the production of high purity isolates appears essential to securing rapid and practical opportunities for change. Likewise, other practical criteria will also be important, such as functionality, price, and sustainability. Indeed, because of extensive processing, the use of chemical and solvents, high energy and water consumption plant protein cracking is less energy efficient than raw and local plant-based foods (52) although some technologies are being developed to address these issues (53). Future works could interestingly use linear programming and minimally processed plant-based foods to assess the added value of protein ingredients compared to raw ingredients (of plant and animal origin) on the quality of protein intake in a complete diet. Constraints could include food amounts, satisfaction of nutritional requirements, price, and climate impact of the diet.

## Conclusion

We conclude that the diverse composition of amino acids from plant protein sources offers simple opportunities to build protein blends that target certain amino acid profiles; these include reference profiles as well as a wide variety of animal proteins. For some more specific animal protein fractions (such as whey protein), perfecting the matches would require expansion of the plant protein portfolio. More generally, the practical nutritional application of plant protein mixes remains limited by the availability of purified ingredients, as most are not readily available at present on the market.

## Data Availability Statement

The original contributions presented in the study are included in the article/Supplementary Material. Further inquiries can be directed to the corresponding author/s.

## Author Contributions

LD was responsible for the study design, data collection, data analysis, and drafting of the manuscript. DR, J-FH, and FM were responsible for support of study design, data analysis, and manuscript drafting. All authors contributed to the article and approved the submitted version.

## Funding

This work was supported by the French Research Agency (ANR), project ANR-18-CE21-0001.

## Conflict of Interest

The authors declare that the research was conducted in the absence of any commercial or financial relationships that could be construed as a potential conflict of interest.

## Publisher's Note

All claims expressed in this article are solely those of the authors and do not necessarily represent those of their affiliated organizations, or those of the publisher, the editors and the reviewers. Any product that may be evaluated in this article, or claim that may be made by its manufacturer, is not guaranteed or endorsed by the publisher.
