# Supplementary material for: Combining Plant Proteins to Achieve Amino Acid Profiles Adapted to Various Nutritional Objectives—An Exploratory Analysis Using Linear Programming
Source: Front Nutr. 2022 Feb 3;8:809685. doi: 10.3389/fnut.2021.809685 (PMC8850771; doi:10.3389/fnut.2021.809685)
Supplement: Supplementary file 1 [file Data_Sheet_1.docx]

Supplementary Material


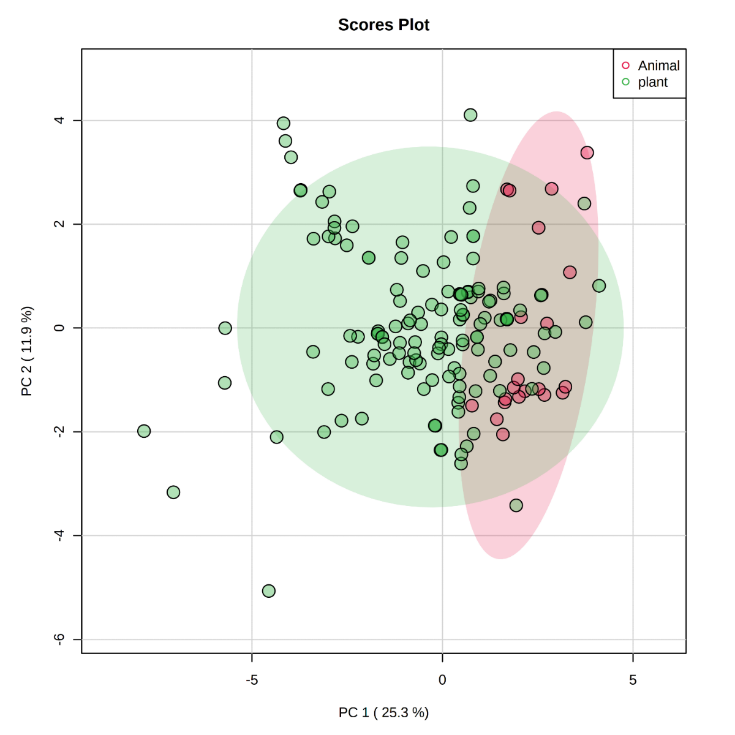
Supplementary Figure 1. Plot of Principal Component Analysis (PCA) scores. Clusters of protein ingredients are based on their source.


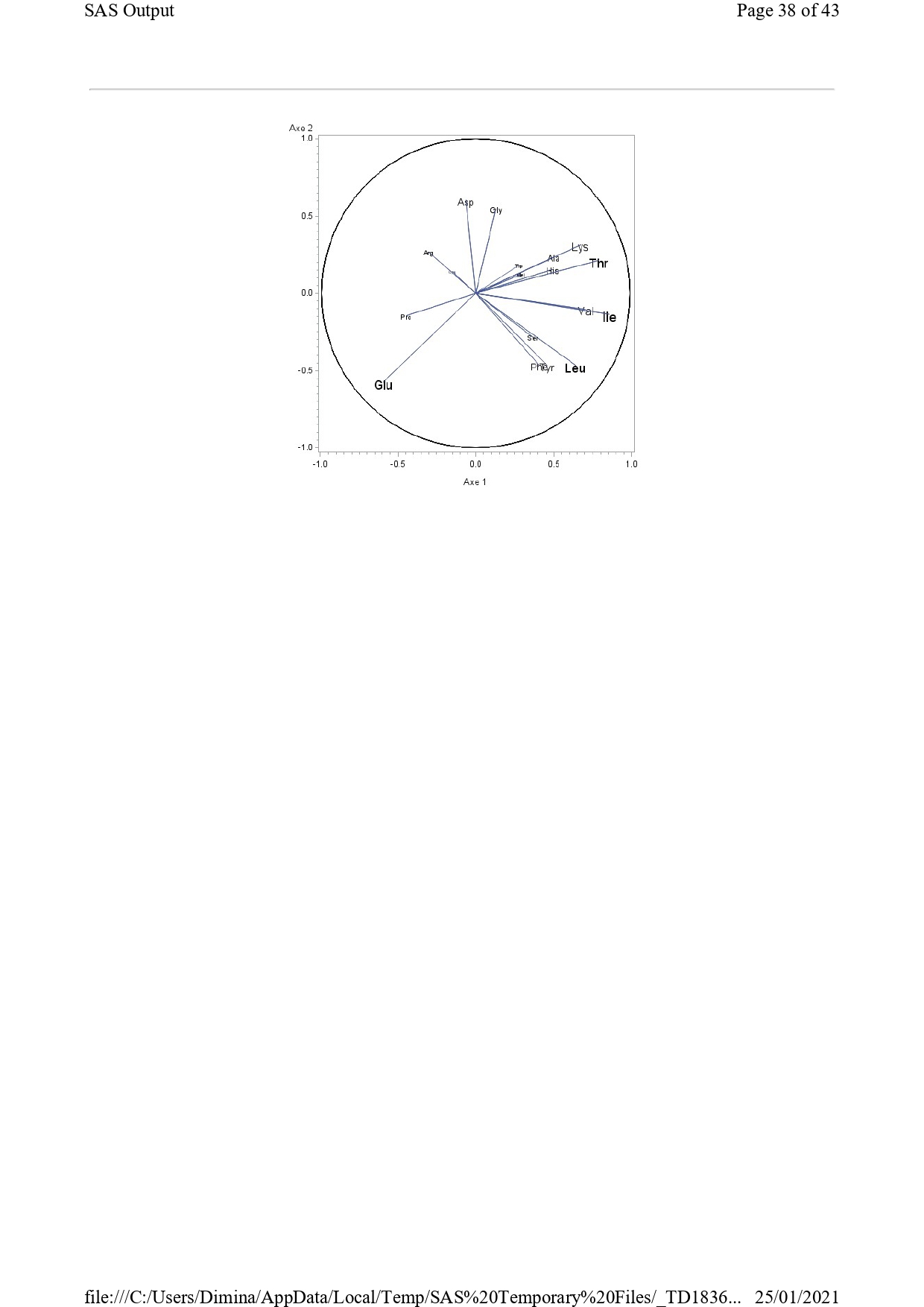
Supplementary Figure 2. PCA/ loading plot. Variables that contribute the most to data variance.

**Supplementary Table 1.** Amino acid Profile 1 - IAA requirements for adults and infants (WHO) used as constraints during optimization.

| IAA | 2007 WHO adult requirements (19,20) | | 2007 WHO infant (0-6 months)  requirements (19,20) | |
| --- | --- | --- | --- | --- |
|  | mg/g | **g/30g** | mg/g | **g/30g** |
| Tryptophan | 15 | **0.18** | 17 | **0.51** |
| Threonine | 30 | **0.69** | 44 | **1.32** |
| Isoleucine | 59 | **0.9** | 55 | **1.65** |
| Leucine | 45 | **1.77** | 96 | **2.88** |
| Lysine | 22 | **1.35** | 69 | **2.07** |
| Met+Cys | 38 | **0.66** | 33 | **0.99** |
| Phe+Tyr | 23 | **1.14** | 94 | **2.82** |
| Valine | 6 | **1.17** | 55 | **1.65** |
| Histidine | 39 | **0.45** | 21 | **0.63** |

**Supplementary Table 2.** Animal profiles added to the database and used as targets for optimization in order to study how plant-based protein isolates can reproduce animal IAA profiles as a function of animal species and meat cuts.

| Animal product | Profile Reference |
| --- | --- |
| Beef: short ribs and sirloin steak | °^1^CIV-INRA |
| Veal: shoulder and chop | °^1^CIV-INRA |
| Lamb: leg and neck | °^1^CIV-INRA |
| Horse: topside and rib steak | °^1^CIV-INRA |
| Pork | ref: 10024 (USDA database) |
| Rabbit | ref: 17180 (USDA database) |
| Chicken | ref: 05011 (USDA database) |
| Turkey | ref: 05167 (USDA database) |
| Duck | ref: 05141 (USDA database) |
| Egg white | ref: 01124 (USDA database) |
| Cow milk | ref: 01151 (USDA database) |
| Acid whey | ref: 01113 (USDA database) |
| Casein | personal data |
| Goat milk | ref: 01106 (USDA database) |
| Sheep milk | ref: 01109 (USDA database) |

^1^ CIV-INRA. Valeurs nutritionnelles des viandes crues. L’essentiel des viandes, 2009

**Supplementary Table 3.** Top 15 protein ingredients with the richest phenylalanine content (in g/100g of amino acids) – protein ingredients of animal and plant origin confounded.

| **Protein ingredients** | **Breadfruit seeds** | **Pigeon peas** | **Macadamia nuts** | **watermelon seed kernels** | **Mung beans** | **Potato Protein Concentrate** | **Alfalfa Protein Concentrate** | **Spearmint** | **Mung beans** | **Navy beans** | **Corn Gluten Meal** | **Yardlong beans** | **Cowpeas catjang** | **Cowpeas common** | **Pinto beans** |
| --- | --- | --- | --- | --- | --- | --- | --- | --- | --- | --- | --- | --- | --- | --- | --- |
| **Data source** | USDA  12001 | USDA  16101 | USDA  12131 | USDA  12174 | USDA  16080 | Personal Data | Personal Data | USDA  02066" | USDA  16083 | USDA  16037 | Personal Data | USDA  16133 | USDA  16060 | USDA  16062 | USDA  16042 |
| **Tryptophan** | 1.47 | 0.98 | 0.70 | 1.23 | 1.12 | 1.54 | 2.05 | 1.82 | 1.07 | 1.27 | 0.52 | 1.24 | 1.24 | 1.24 | 1.27 |
| **Threonine** | 4.59 | 3.54 | 3.84 | 3.50 | 3.38 | 5.81 | 4.93 | 4.85 | 3.57 | 3.65 | 3.20 | 3.84 | 3.84 | 3.84 | 4.34 |
| **Isoleucine** | 5.28 | 3.62 | 3.26 | 4.22 | 4.36 | 5.27 | 5.34 | 4.85 | 5.25 | 4.89 | 3.83 | 4.10 | 4.10 | 4.10 | 4.67 |
| **Leucine** | 6.71 | 7.15 | 6.25 | 6.76 | 7.99 | 9.65 | 9.65 | 8.89 | 8.52 | 8.84 | 15.35 | 7.73 | 7.73 | 7.73 | 8.35 |
| **Lysine** | 6.79 | 7.02 | 0.19 | 2.79 | 7.20 | 7.49 | 6.37 | 5.10 | 6.83 | 6.57 | 1.63 | 6.82 | 6.83 | 6.82 | 7.27 |
| **Methionine** | 1.14 | 1.12 | 0.24 | 2.62 | 1.24 | 2.17 | 2.05 | 1.67 | 1.50 | 1.40 | 2.22 | 1.43 | 1.44 | 1.44 | 1.39 |
| **Cystine** | 1.38 | 1.15 | 0.06 | 1.38 | 0.91 | 1.33 | 1.03 | 1.31 | 0.95 | 0.96 | 1.86 | 1.12 | 1.11 | 1.12 | 1.00 |
| **Phenylalanine** | 9.50 | 8.58 | 6.90 | 6.39 | 6.24 | 6.24 | 6.16 | 6.04 | 6.01 | 5.94 | 5.91 | 5.89 | 5.89 | 5.89 | 5.87 |
| **Tyrosine** | 6.48 | 2.48 | 5.31 | 3.19 | 3.09 | 5.41 | 4.52 | 3.56 | 3.20 | 2.48 | 4.63 | 3.26 | 3.26 | 3.26 | 2.29 |
| **Valine** | 6.38 | 4.32 | 3.77 | 4.89 | 5.35 | 6.31 | 6.37 | 5.91 | 5.78 | 6.37 | 4.34 | 4.81 | 4.81 | 4.81 | 5.35 |
| **Arginine** | 5.89 | 6.00 | 14.56 | 15.40 | 7.23 | 4.87 | 6.37 | 5.45 | 6.70 | 5.23 | 3.01 | 6.99 | 6.99 | 6.99 | 5.87 |
| **Histidine** | 2.47 | 3.57 | 2.02 | 2.44 | 3.01 | 2.16 | 2.46 | 2.37 | 2.88 | 2.60 | 2.15 | 3.13 | 3.13 | 3.13 | 2.98 |
| **Alanine** | 4.00 | 4.49 | 4.03 | 4.69 | 4.54 | 4.89 | 6.37 | 6.15 | 4.39 | 4.66 | 8.12 | 4.60 | 4.60 | 4.60 | 4.67 |
| **Aspartate** | 9.74 | 9.90 | 11.41 | 8.69 | 11.92 | 12.09 | 10.27 | 13.98 | 12.03 | 13.33 | 5.52 | 12.18 | 12.18 | 12.18 | 12.16 |
| **Glutamate** | 12.35 | 23.22 | 23.54 | 17.92 | 18.45 | 10.28 | 11.50 | 12.90 | 16.84 | 15.90 | 20.71 | 19.11 | 19.11 | 19.10 | 16.23 |
| **Glycine** | 5.54 | 3.70 | 4.71 | 5.23 | 4.13 | 4.68 | 5.54 | 5.68 | 4.30 | 4.11 | 2.68 | 4.16 | 4.17 | 4.16 | 4.27 |
| **Proline** | 4.40 | 4.41 | 4.86 | 3.93 | 4.74 | 4.67 | 4.72 | 4.85 | 4.76 | 5.73 | 9.48 | 4.54 | 4.53 | 4.53 | 5.75 |
| **Serine** | 5.91 | 4.74 | 4.35 | 4.74 | 5.09 | 5.15 | 4.31 | 4.62 | 5.42 | 6.06 | 4.84 | 5.05 | 5.05 | 5.05 | 6.28 |
| **Total AA (g)** | 100 | 100 | 100 | 100.00 | 100.00 | 100.00 | 100.00 | 100.00 | 100.00 | 100.00 | 100.00 | 100.00 | 100.00 | 100.00 | 100.00 |


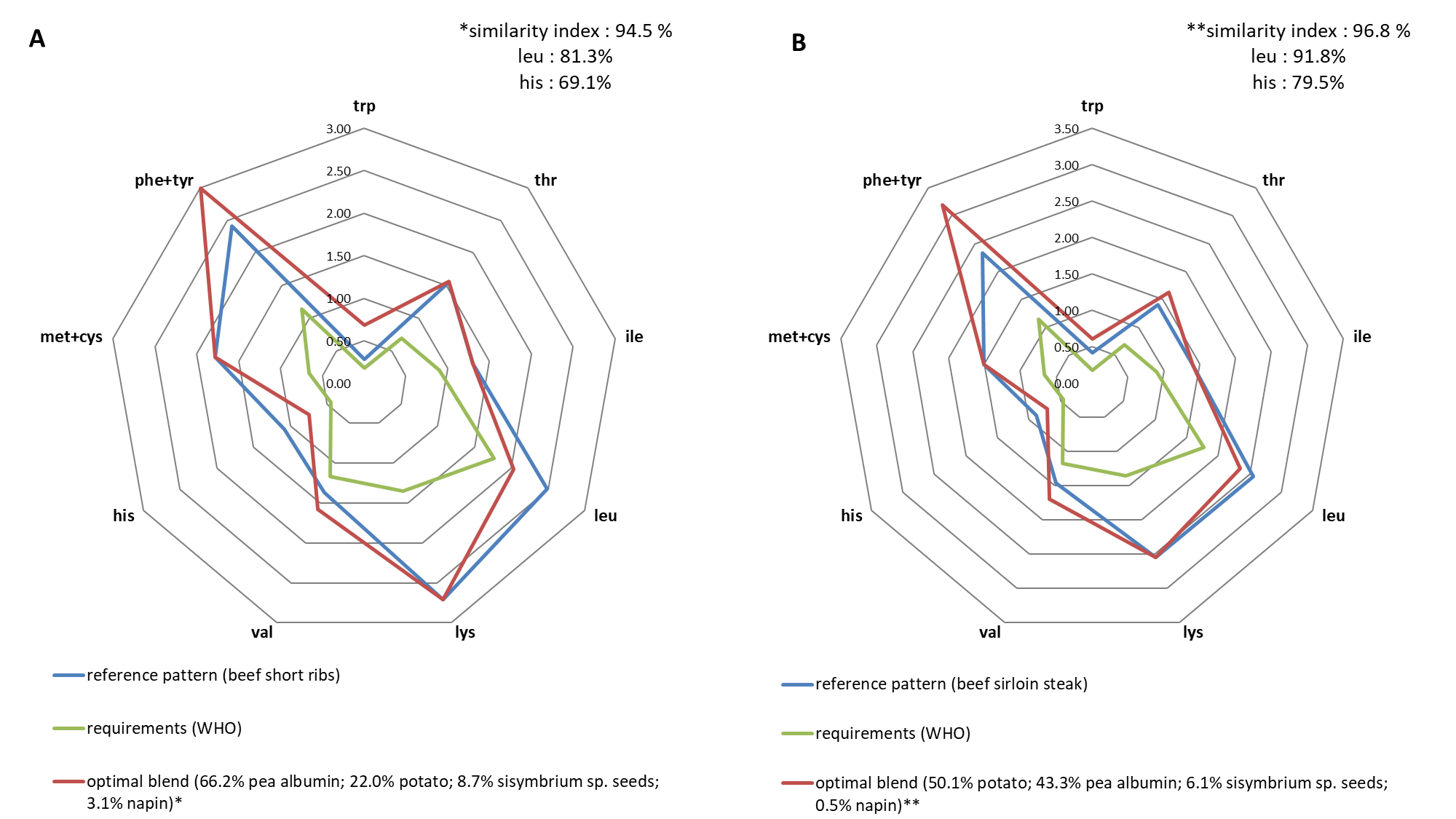
**Supplementary Figure 3.** IAA profiles (g/30g) of plant blends that best replicate the IAA profiles of beef short ribs (panel A) and beef sirloin steak (panel B).


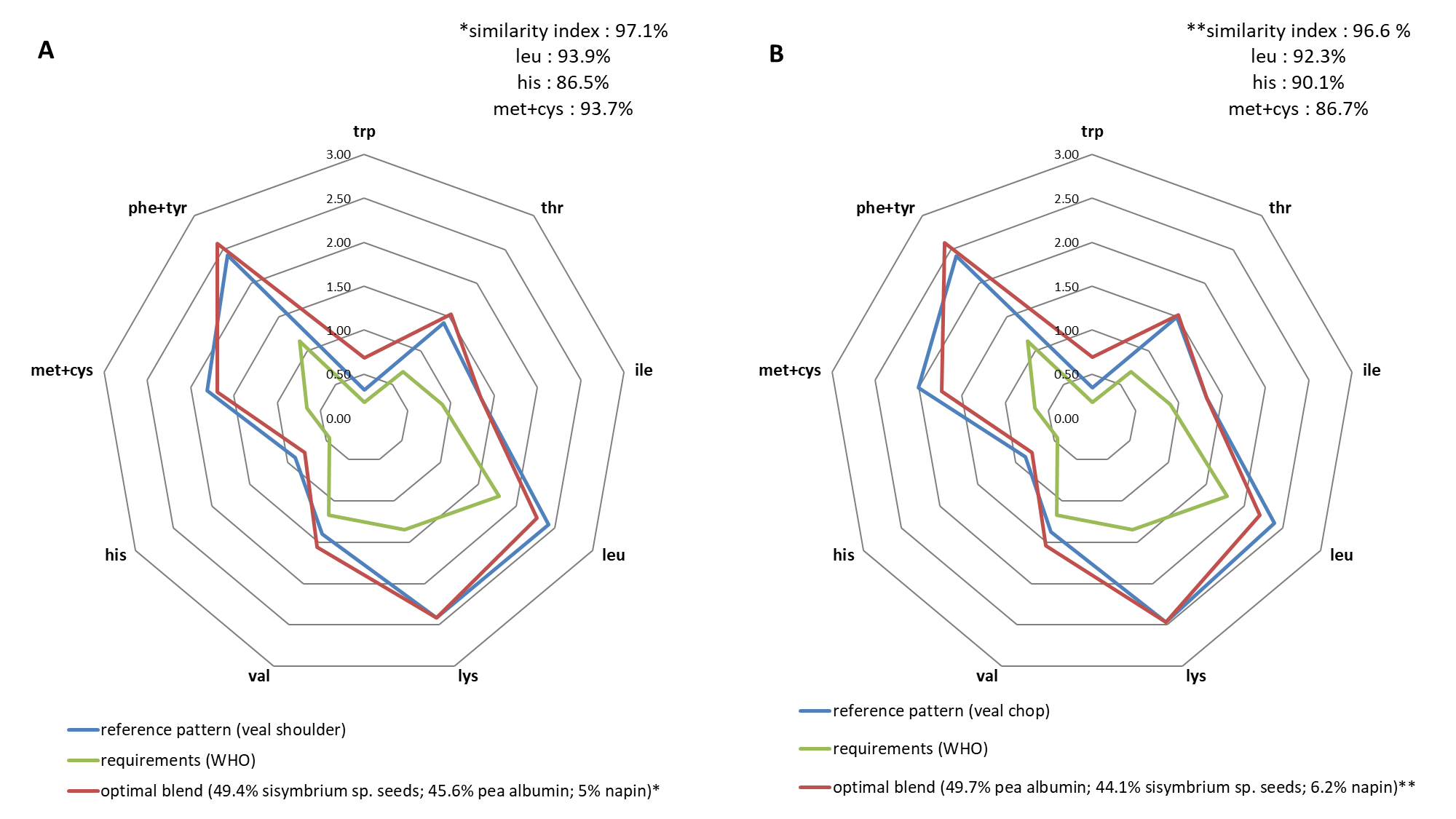
**Supplementary Figure 4.** IAA profiles (g/30g) of plant blends that best replicate the IAA profiles of veal shoulder (panel A) and veal chop (panel B).


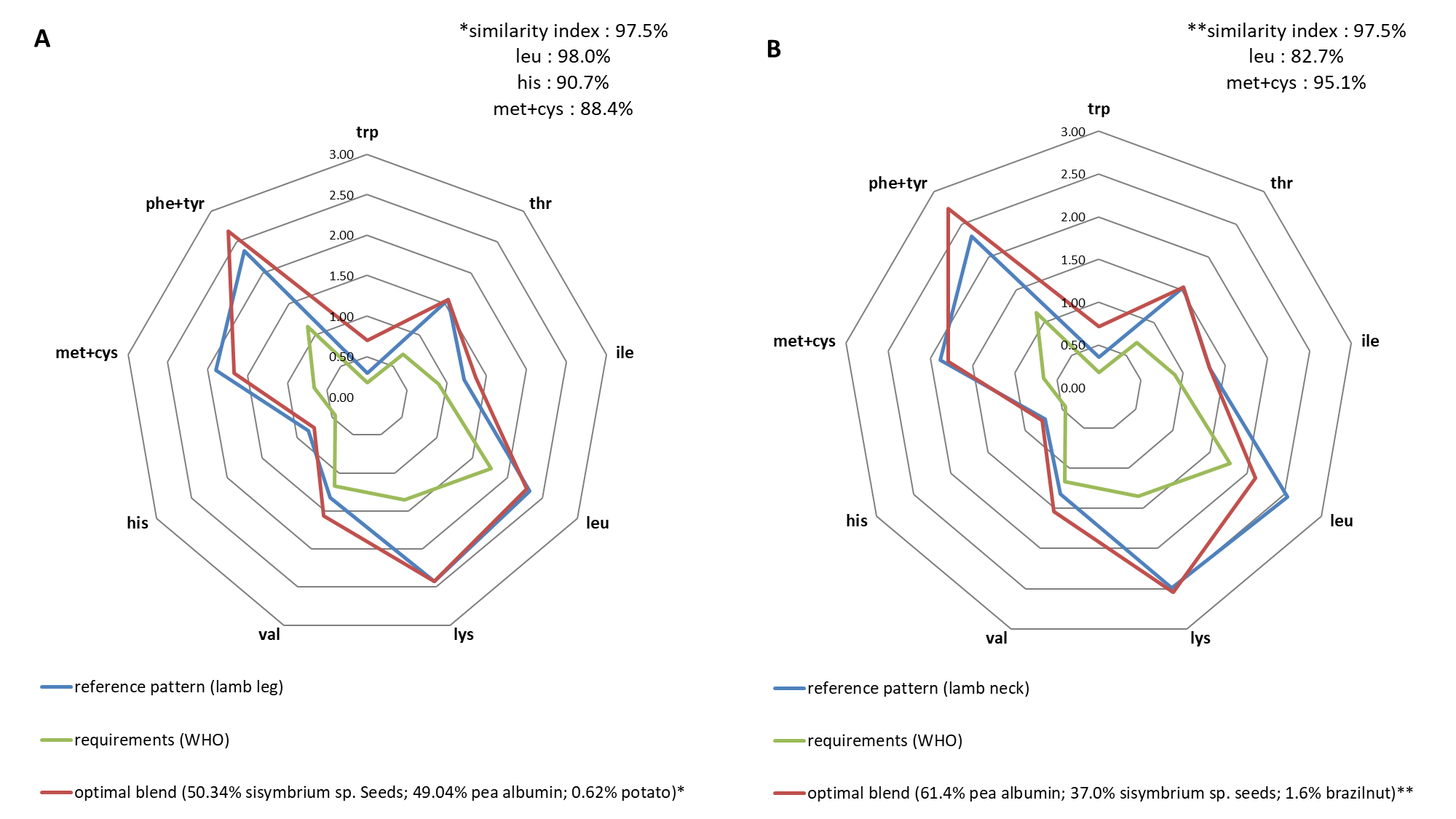
**Supplementary Figure 5.** IAA profiles (g/30g) of plant blends that best replicate the IAA profiles of lamb leg (panel A) and lamb neck (panel B).


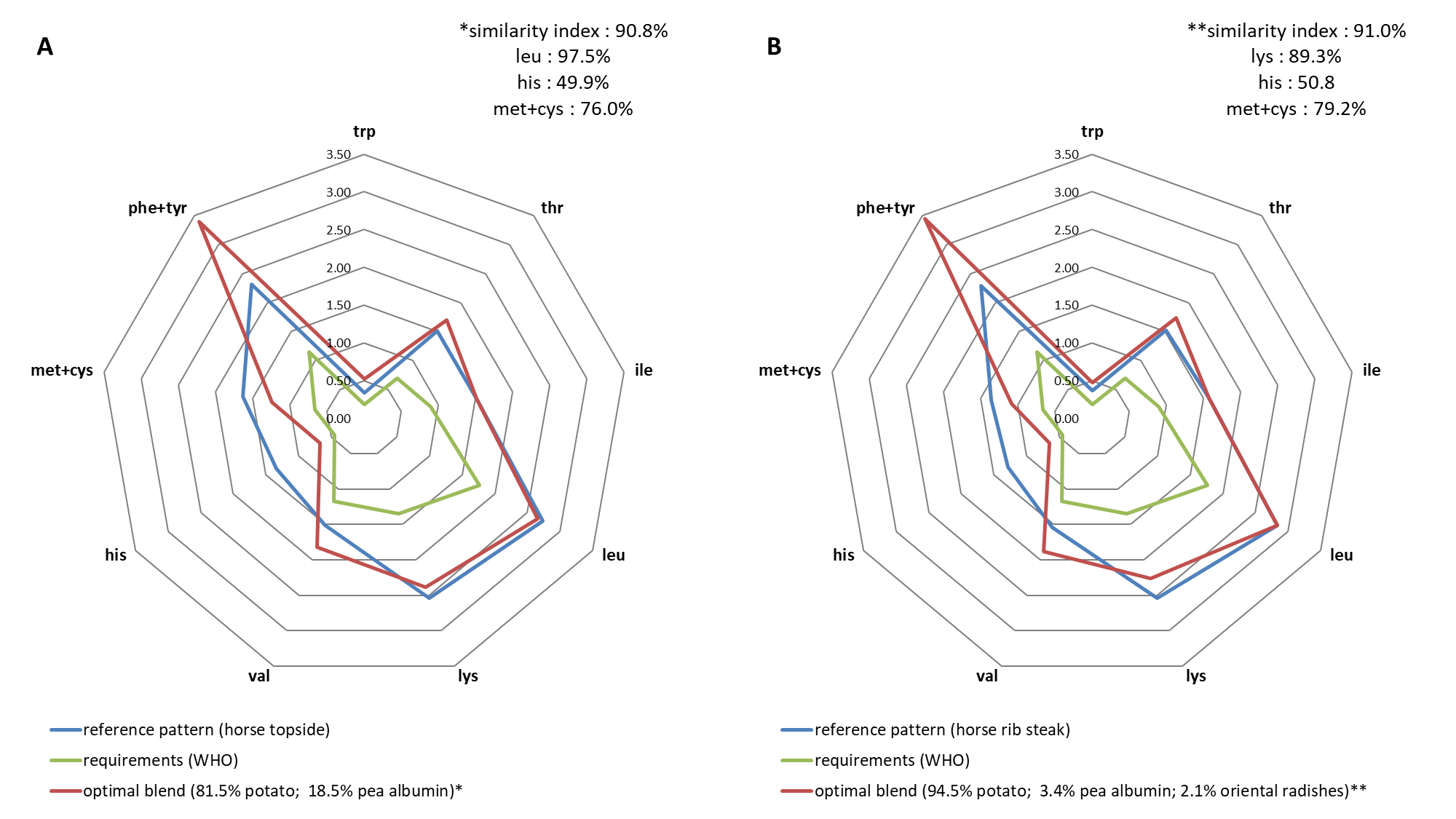
**Supplementary Figure 6.** IAA profiles (g/30g) of plant blends that best replicate the IAA profiles of horse topside (panel A) and horse rib steak (panel B).


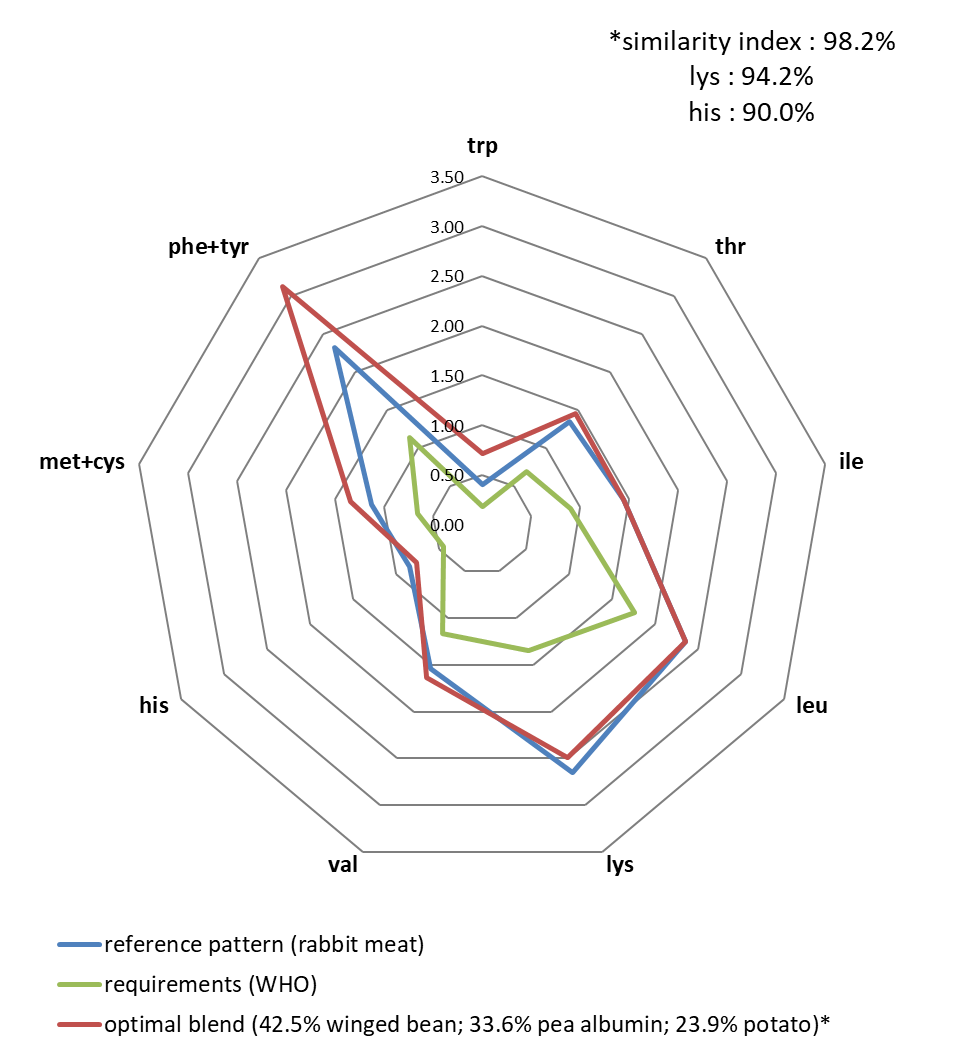
 Supplementary Figure 7. IAA profiles (g/30g) of plant blends that best replicate the IAA profile of rabbit.


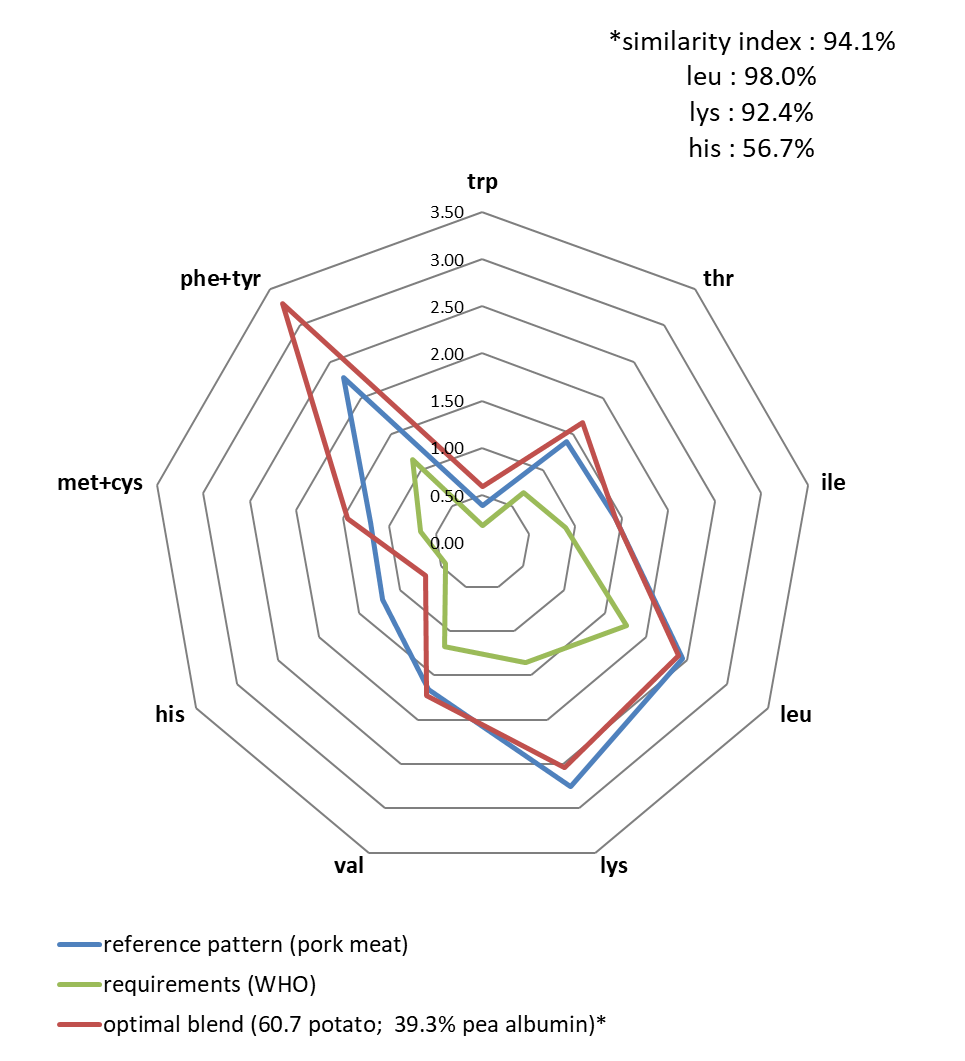
 **Supplementary Figure 8.** IAA profiles (g/30g) of plant blends that best replicate the IAA profile of pork.


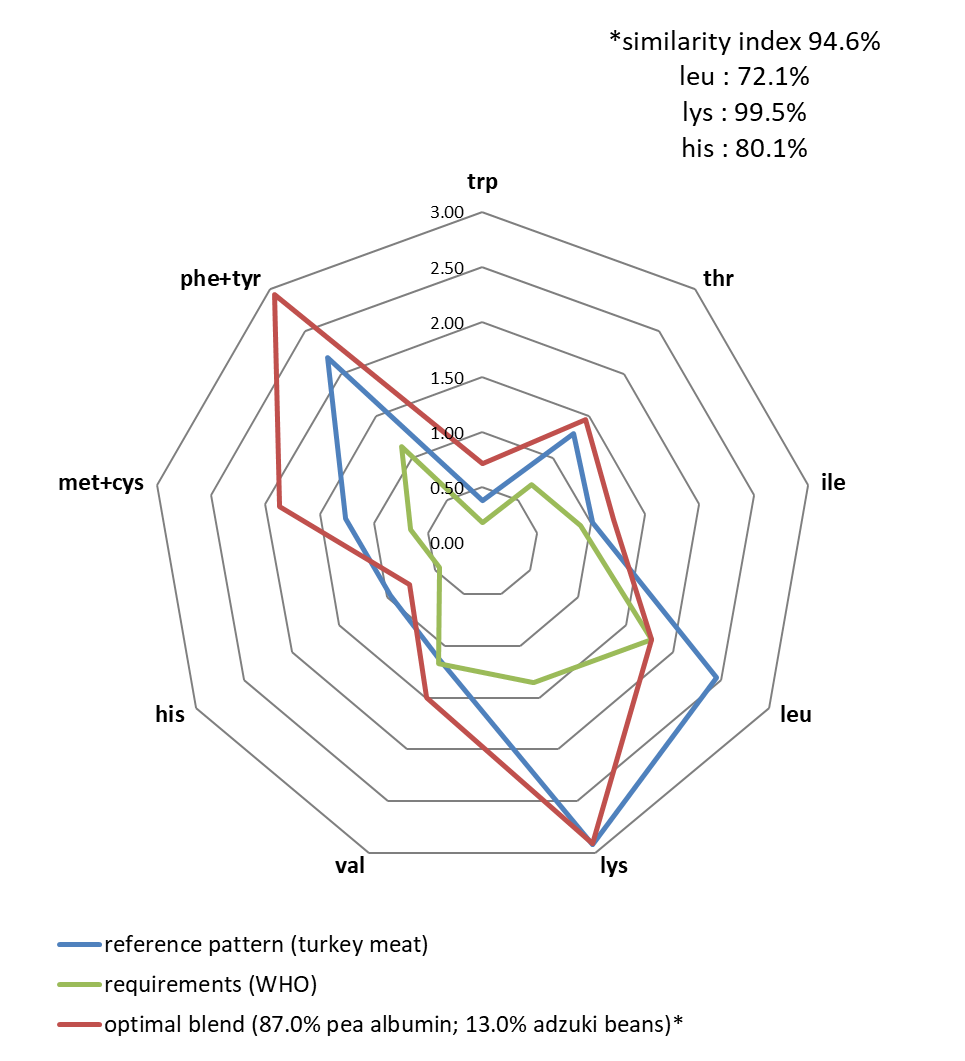
 **Supplementary Figure 9.** IAA profiles (g/30g) of plant blends that best replicate the IAA profile of turkey.


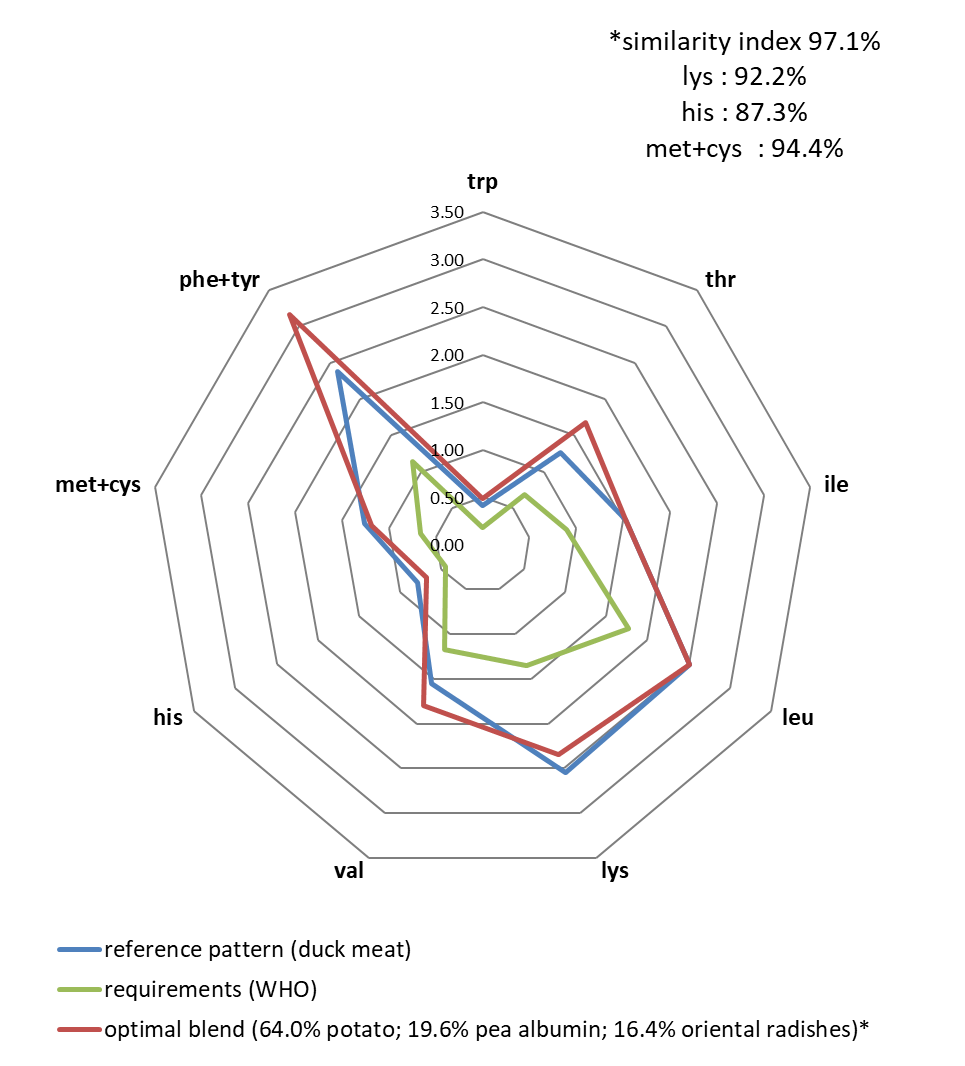
 **Supplementary Figure 10.** IAA profiles (g/30g) of plant blends that best replicate the IAA profile of duck.


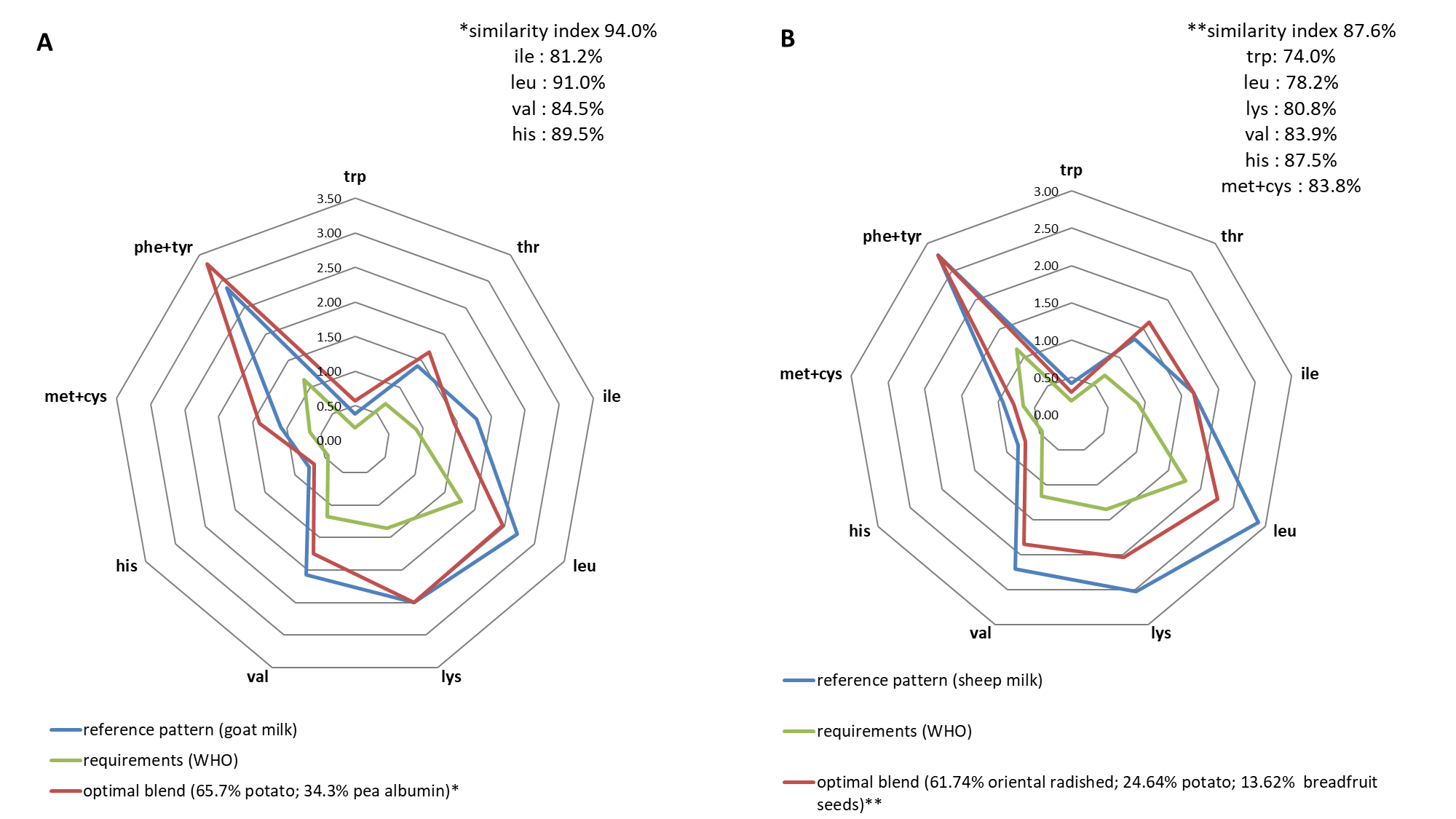
**Supplementary Figure 11.** IAA profiles (g/30g) of plant blends that best replicate the IAA profile of total goat milk (panel A) and total sheep milk (panel B).


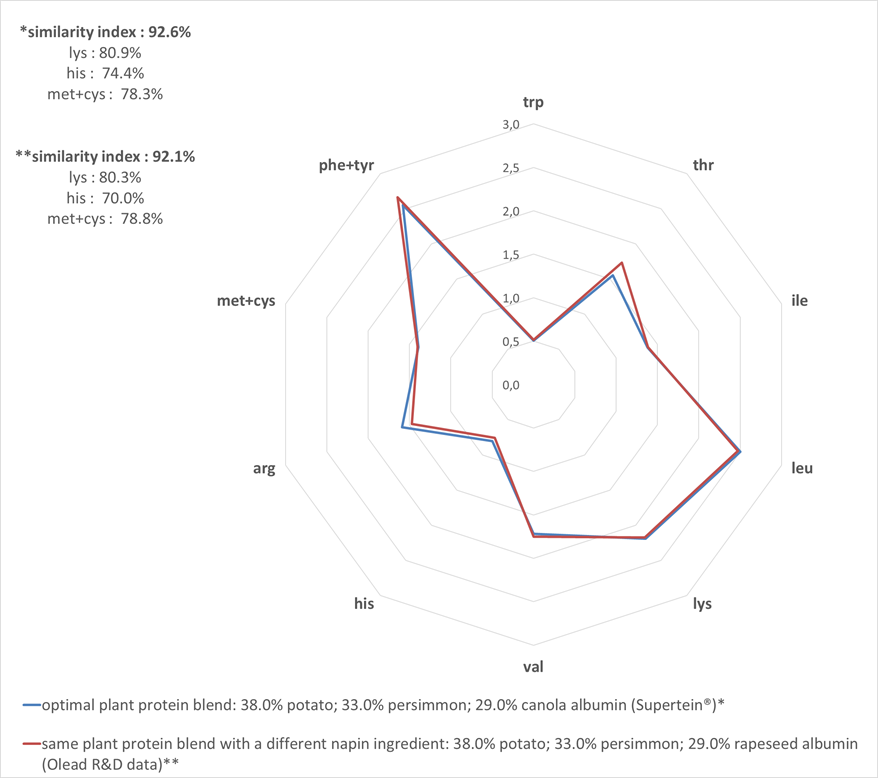
**Supplementary Figure 12.** variability of IAA profiles according to the origin of the rapeseed/ canola albumin fraction.

**Supplementary Table 4.** Active constraints during optimization that rendered the problems unworkable and led to unmet non-active constraints. Active constraints needed to be relaxed and any violation of the constraints was minimized by optimization.

| Target AA profile | Active constraints* | Unmet constraints |
| --- | --- | --- |
| ‘animal’: beef short ribs | his, leu | his, leu |
| ‘animal’: beef sirloin steak | lys, leu | leu, his |
| ‘animal’: veal shoulder | ile, cys+met | leu, his, cys+met |
| ‘animal’: veal chop | ile, met+cys | leu, his, cys+met |
| ‘animal’: lamb leg | thr, met+cys | leu, his, cys+met |
| ‘animal’: lamb neck | thr, leu, met+cys | cys+met |
| ‘animal’: horse topside | his | leu, lys, met+cys, his |
| ‘animal’: horse rib steak | lys, leu | his, met+cys, lys |
| ‘animal’: rabbit | leu, lys | His, lys |
| ‘animal’: pork | his | leu, lys, his |
| ‘animal’: chicken | lys, ile | trp, leu, his, met+cys, lys |
| ‘animal’: turkey | lys, his | leu, lys, his |
| ‘animal’: duck | ile, lys | his, met+cys, lys |
| ‘animal’: egg white | ile, met+cys | val, ile |
| ‘animal’: breast milk | met+cys, ile | Vvl, ile |
| ‘animal’: cow milk | his, leu, lys | his |
| ‘animal’: whey | leu, lys | trp, met+cys, leu, lys |
| ‘animal’: casein | leu, lys | his, leu |
| ‘animal’: goat milk | ile | leu, val, his, ile |
| ‘animal’: sheep milk | ile, lys | trp, leu, val, his, met+cys, lys |

* By definition, the weight of the blend is always an active constraint.

**Supplementary Table 5.** amino acid composition (g/30g) of animal products and the plant blends that best mimicked their composition.

|  | Amino acid content (g / 30g protein) | | | | | | | | | | | | | | | | | |
| --- | --- | --- | --- | --- | --- | --- | --- | --- | --- | --- | --- | --- | --- | --- | --- | --- | --- | --- |
|  | **trp** | **thr** | **ile** | **leu** | **lys** | **met** | **cys** | **phe** | **tyr** | **val** | arg | **his** | ala | asp | glu | gly | pro | ser |
| 1: Beef short ribs AA profile | 0.28 | 1.55 | 1.30 | 2.48 | 2.71 | 1.27 | 0.51 | 1.34 | 1.07 | 1.37 | 1.90 | 1.08 | 1.73 | 2.81 | 4.92 | 1.34 | 1.12 | 1.20 |
| Optimal plant blend replicating 1 | 0.69 | 1.56 | 1.30 | **2.03^1^** | 2.71 | 0.54 | 1.24 | 1.71 | 1.29 | 1.58 | 1.53 | **0.75** | 2.18 | 3.36 | 2.97 | 1.74 | 1.82 | 1.01 |
| 2: Beef sirloin steak AA profile | 0.42 | 1.40 | 1.41 | 2.55 | 2.55 | 1.06 | 0.45 | 1.29 | 1.04 | 1.45 | 1.93 | 0.89 | 1.81 | 2.61 | 4.83 | 1.73 | 1.35 | 1.21 |
| Optimal plant blend replicating 2 | 0.61 | 1.63 | 1.41 | **2.35** | 2.55 | 0.57 | 0.93 | 1.78 | 1.42 | 1.69 | 1.50 | **0.71** | 1.94 | 3.50 | 2.94 | 1.63 | 1.65 | 1.20 |
| 3: veal shoulder AA profile | 0.32 | 1.41 | 1.35 | 2.42 | 2.41 | 1.10 | 0.71 | 1.31 | 1.11 | 1.40 | 1.94 | 0.90 | 1.90 | 2.74 | 4.79 | 1.70 | 1.34 | 1.16 |
| Optimal plant blend replicating 3 | 0.69 | 1.54 | 1.35 | **2.27** | 2.41 | **0.61** | **1.08** | 1.51 | 1.08 | 1.56 | 1.70 | **0.78** | 2.01 | 3.30 | 3.60 | 1.74 | 1.81 | 0.98 |
| 4: veal chop AA profile | 0.34 | 1.50 | 1.33 | 2.39 | 2.46 | 1.29 | 0.71 | 1.33 | 1.07 | 1.37 | 2.00 | 0.87 | 1.75 | 2.78 | 4.89 | 1.52 | 1.21 | 1.20 |
| Optimal plant blend replicating 4 | 0.69 | 1.53 | 1.33 | **2.21** | 2.46 | **0.60** | **1.14** | 1.52 | 1.08 | 1.55 | 1.68 | **0.78** | 2.05 | 3.26 | 3.58 | 1.74 | 1.84 | 0.97 |
| 5: lamb leg AA profile | 0.30 | 1.56 | 1.21 | 2.32 | 2.43 | 1.23 | 0.66 | 1.35 | 1.01 | 1.32 | 1.99 | 0.84 | 1.93 | 2.84 | 4.82 | 1.73 | 1.22 | 1.23 |
| Optimal plant blend replicating 5 | 0.70 | 1.56 | 1.36 | **2.27** | 2.43 | 0.60 | 1.08 | 1.55 | 1.12 | 1.57 | **1.68** | 0.76 | 2.06 | 3.43 | 3.35 | 1.76 | 1.75 | 0.98 |
| 6: lamb neck AA profile | 0.35 | 1.54 | 1.31 | 2.55 | 2.49 | 1.11 | 0.78 | 1.27 | 1.04 | 1.32 | 2.09 | 0.73 | 1.87 | 2.77 | 4.11 | 2.06 | 1.40 | 1.22 |
| Optimal plant blend replicating 6 | 0.71 | 1.54 | 1.31 | **2.11** | 2.55 | 0.59 | 1.20 | 1.59 | 1.14 | 1.54 | 1.67 | 0.76 | 2.17 | 3.40 | 3.20 | 1.79 | 1.79 | 0.94 |
| 7: horse topside AA profile | 0.34 | 1.51 | 1.51 | 2.73 | 2.54 | 1.14 | 0.49 | 1.28 | 1.04 | 1.51 | 1.78 | 1.34 | 1.77 | 2.84 | 4.60 | 1.33 | 1.10 | 1.14 |
| Optimal plant blend replicating 7 | 0.52 | 1.70 | 1.51 | **2.66** | **2.38** | 0.62 | 0.62 | 1.85 | 1.55 | 1.82 | 1.47 | **0.67** | 1.66 | 3.58 | 2.98 | 1.49 | 1.50 | 1.41 |
| 8: horse rib steak AA profile | 0.37 | 1.52 | 1.57 | 2.83 | 2.54 | 0.96 | 0.40 | 1.28 | 1.02 | 1.54 | 1.83 | 1.29 | 1.73 | 2.74 | 4.61 | 1.44 | 1.20 | 1.14 |
| Optimal plant blend replicating 8 | 0.47 | 1.73 | 1.57 | 2.83 | **2.27** | **0.64** | **0.44** | 1.85 | 1.59 | 1.88 | 1.48 | **0.65** | 1.50 | 3.60 | 3.16 | 1.42 | 1.41 | 1.51 |
| 9: pork meat AA profile | 0.39 | 1.39 | 1.43 | 2.45 | 2.75 | 0.81 | 0.39 | 1.22 | 1.06 | 1.66 | 1.90 | 1.22 | 1.78 | 2.83 | 4.78 | 1.45 | 1.23 | 1.26 |
| Optimal plant blend replicating 9 | 0.59 | 1.65 | 1.43 | **2.40** | **2.54** | 0.58 | 0.87 | 1.82 | 1.48 | 1.73 | 1.47 | **0.69** | 1.89 | 3.53 | 2.87 | 1.59 | 1.61 | 1.26 |
| 10: rabbit meat AA profile | 0.40 | 1.35 | 1.44 | 2.36 | 2.65 | 0.76 | 0.38 | 1.24 | 1.08 | 1.54 | 1.87 | 0.85 | 1.83 | 2.96 | 4.85 | 1.64 | 1.48 | 1.34 |
| Optimal plant blend replicating 10 | 0.71 | 1.45 | 1.44 | 2.36 | **2.49** | 0.47 | 0.88 | 1.67 | 1.46 | 1.63 | 1.69 | **0.76** | 1.67 | 3.42 | 3.38 | 1.48 | 1.84 | 1.20 |
| 11: chicken meat AA profile | 0.37 | 1.35 | 1.69 | 2.40 | 2.71 | 0.88 | 0.41 | 1.27 | 1.08 | 1.58 | 1.92 | 0.99 | 1.74 | 2.85 | 4.78 | 1.57 | 1.31 | 1.10 |
| Optimal plant blend replicating 11 | **0.29** | 1.63 | 1.69 | **2.23** | **2.02** | **0.42** | **0.34** | 1.41 | 0.93 | 1.82 | 2.13 | **0.73** | 1.27 | 2.88 | 6.57 | 1.26 | 1.13 | 1.26 |
| 12: turkey meat AA profile | 0.38 | 1.29 | 1.02 | 2.46 | 2.92 | 0.93 | 0.33 | 1.14 | 1.05 | 1.13 | 2.01 | 0.96 | 1.92 | 2.83 | 4.81 | 1.51 | 1.91 | 1.40 |
| Optimal plant blend replicating 12 | 0.72 | 1.46 | 1.21 | **1.77** | **2.91** | 0.45 | 1.43 | 1.73 | 1.21 | 1.49 | 1.56 | **0.77** | 2.44 | 3.42 | 2.86 | 1.80 | 1.86 | 0.91 |
| 13: duck meat AA profile | 0.41 | 1.27 | 1.53 | 2.51 | 2.54 | 0.80 | 0.46 | 1.25 | 1.13 | 1.56 | 1.90 | 0.79 | 1.88 | 2.91 | 4.65 | 1.67 | 1.46 | 1.28 |
| Optimal plant blend replicating 13 | 0.48 | 1.68 | 1.53 | 2.51 | **2.35** | 0.57 | 0.62 | 1.75 | 1.41 | 1.80 | 1.60 | **0.69** | 1.64 | 3.42 | 3.69 | 1.47 | 1.44 | 1.34 |
| 14: egg white AA profile | 0.32 | 1.15 | 1.69 | 2.59 | 2.06 | 1.02 | 0.73 | 1.75 | 1.17 | 2.07 | 1.65 | 0.74 | 1.80 | 3.11 | 3.96 | 1.05 | 1.11 | 2.04 |
| Optimal plant blend replicating 14 | 0.50 | 1.33 | **1.24** | 2.59 | 2.06 | 0.76 | 0.99 | 1.66 | 1.26 | **1.53** | 1.69 | 0.74 | 2.03 | 2.84 | 4.14 | 1.48 | 1.98 | 1.18 |
| 15: breast milk AA profile | 0.52 | 1.40 | 1.70 | 2.89 | 2.07 | 0.64 | 0.58 | 1.40 | 1.61 | 1.91 | 1.31 | 0.70 | 1.09 | 2.49 | 5.11 | 0.79 | 2.49 | 1.31 |
| Optimal plant blend replicating 15 | 0.52 | 1.64 | **1.52** | 2.89 | 2.07 | 0.69 | 0.52 | 1.66 | **1.35** | 1.76 | 1.63 | 0.70 | 1.58 | 3.45 | 3.64 | 1.48 | 1.55 | 1.36 |
| 16: cow milk AA profile | 0.36 | 1.20 | 1.45 | 2.66 | 2.35 | 0.73 | 0.18 | 1.46 | 1.42 | 1.84 | 0.80 | 0.85 | 0.95 | 2.40 | 6.32 | 0.56 | 2.77 | 1.69 |
| Optimal plant blend replicating 16 | 0.46 | 1.22 | 1.45 | 2.66 | 2.35 | 0.35 | 0.55 | 1.74 | 1.25 | 1.88 | 1.83 | **0.76** | 1.41 | 3.62 | 4.02 | 1.39 | 1.64 | 1.42 |
| 17: cow acid whey AA profile | 0.66 | 1.61 | 1.59 | 3.05 | 2.75 | 0.60 | 0.58 | 1.05 | 0.82 | 1.58 | 0.89 | 0.63 | 1.38 | 3.14 | 5.72 | 0.58 | 1.91 | 1.48 |
| Optimal plant blend replicating 17 | **0.45** | 1.74 | 1.59 | **2.86** | **2.23** | **0.64** | **0.40** | 1.85 | 1.59 | 1.89 | 1.49 | 0.65 | 1.46 | 3.59 | 3.27 | 1.40 | 1.39 | 1.53 |
| 18: cow casein AA profile | 0.39 | 1.23 | 1.53 | 2.88 | 2.37 | 0.75 | 0.21 | 1.38 | 1.44 | 1.86 | 0.99 | 0.78 | 0.93 | 2.19 | 6.30 | 0.54 | 2.79 | 1.44 |
| Optimal plant blend replicating 18 | 0.42 | 1.28 | 1.53 | **2.72** | 2.37 | 0.34 | 0.62 | 1.88 | 1.32 | 2.10 | 1.69 | **0.68** | 1.31 | 3.76 | 3.45 | 1.53 | 1.58 | 1.43 |
| 19: goat milk AA profile | 0.38 | 1.41 | 1.78 | 2.71 | 2.50 | 0.69 | 0.40 | 1.34 | 1.54 | 2.07 | 1.03 | 0.77 | 1.02 | 1.81 | 5.40 | 0.43 | 3.17 | 1.56 |
| Optimal plant blend replicating 19 | 0.57 | 1.67 | **1.45** | **2.46** | 2.50 | 0.59 | 0.81 | 1.83 | 1.50 | **1.75** | 1.47 | **0.69** | 1.83 | 3.54 | 2.90 | 1.57 | 1.58 | 1.29 |
| 20: sheep milk AA profile | 0.41 | 1.32 | 1.67 | 2.89 | 2.53 | 0.76 | 0.17 | 1.40 | 1.38 | 2.21 | 0.98 | 0.82 | 1.33 | 1.62 | 5.02 | 0.20 | 2.86 | 2.42 |
| Optimal plant blend replicating 20 | **0.31** | 1.62 | 1.67 | **2.26** | **2.04** | **0.44** | **0.35** | 1.66 | 1.13 | **1.85** | 2.01 | **0.72** | 1.29 | 2.95 | 5.87 | 1.34 | 1.15 | 1.36 |
